# *BRCA1* and *BRCA2* 5′ noncoding region variants identified in breast cancer patients alter promoter activity and protein binding

**DOI:** 10.1002/humu.23652

**Published:** 2018-09-24

**Authors:** Leslie J. Burke, Jan Sevcik, Gaetana Gambino, Emma Tudini, Eliseos J. Mucaki, Ben C. Shirley, Phillip Whiley, Michael T. Parsons, Kim De Leeneer, Sara Gutiérrez‐Enríquez, Marta Santamariña, Sandrine M. Caputo, Elizabeth Santana dos Santos, Jana Soukupova, Marketa Janatova, Petra Zemankova, Klara Lhotova, Lenka Stolarova, Mariana Borecka, Alejandro Moles‐Fernández, Siranoush Manoukian, Bernardo Bonanni, ENIGMA Consortium, Stacey L. Edwards, Marinus J. Blok, Thomas van Overeem Hansen, Maria Rossing, Orland Diez, Ana Vega, Kathleen B.M. Claes, David E. Goldgar, Etienne Rouleau, Paolo Radice, Paolo Peterlongo, Peter K. Rogan, Maria Caligo, Amanda B. Spurdle, Melissa A. Brown

**Affiliations:** ^1^ School of Chemistry and Molecular Biosciences University of Queensland Brisbane Australia; ^2^ Institute of Biochemistry and Experimental Oncology, First Faculty of Medicine Charles University Prague Czech Republic; ^3^ Section of Molecular Genetics Department of Laboratory Medicine University Hospital of Pisa Pisa Italy; ^4^ Department of Genetics and Computational Biology QIMR Berghofer Medical Research Institute Brisbane Australia; ^5^ University of Western Ontario, Department of Biochemistry Schulich School of Medicine and Dentistry London Ontario Canada; ^6^ CytoGnomix Inc. London Ontario Canada; ^7^ Center for Medical Genetics Ghent University Hospital and Cancer Research Institute Ghent (CRIG) Ghent University Ghent Belgium; ^8^ Oncogenetics Group Vall d'Hebron Institute of Oncology (VHIO) Barcelona Spain; ^9^ Fundación Pública Galega de Medicina Xenómica‐SERGAS Grupo de Medicina Xenómica‐USC, CIBERER, IDIS Santiago de Compostela Spain; ^10^ Service de Génétique Department de Biologie des Tumeurs Institut Curie Paris France; ^11^ Department of oncology Center for Translational Oncology Cancer Institute of the State of São Paulo ‐ ICESP São Paulo Brazil; ^12^ A.C.Camargo Cancer Center São Paulo Brazil; ^13^ Unit of Medical Genetics Department of Medical Oncology and Hematology Fondazione IRCCS (Istituto di Ricovero e Cura a Carattere Scientifico) Istituto Nazionale dei Tumori (INT) Milan Italy; ^14^ Division of Cancer Prevention and Genetics Istituto Europeo di Oncologia Milan Italy; ^15^ Department of Clinical Genetics Maastricht University Medical Centre Maastricht The Netherlands; ^16^ Center for Genomic Medicine Copenhagen University Hospital, Rigshospitalet Copenhagen Denmark; ^17^ Area of Clinical and Molecular Genetics University Hospital Vall d'Hebron (UHVH) Barcelona Spain; ^18^ Huntsman Cancer Institute University of Utah Salt Lake City Utah; ^19^ Gustave Roussy Villejuif France; ^20^ Unit of Molecular Bases of Genetic Risk and Genetic Testing Department of Research Fondazione IRCCS Istituto Nazionale dei Tumori di Milano Milan Italy; ^21^ IFOM Fondazione Istituto FIRC di Oncologia Molecolare Milan Italy

**Keywords:** breast cancer, BRCA1, BRCA2, promoter, transcription, variants of unknown clinical significance (VUS)

## Abstract

The widespread use of next generation sequencing for clinical testing is detecting an escalating number of variants in noncoding regions of the genome. The clinical significance of the majority of these variants is currently unknown, which presents a significant clinical challenge. We have screened over 6,000 early‐onset and/or familial breast cancer (BC) cases collected by the ENIGMA consortium for sequence variants in the 5′ noncoding regions of BC susceptibility genes *BRCA1* and *BRCA2*, and identified 141 rare variants with global minor allele frequency < 0.01, 76 of which have not been reported previously. Bioinformatic analysis identified a set of 21 variants most likely to impact transcriptional regulation, and luciferase reporter assays detected altered promoter activity for four of these variants. Electrophoretic mobility shift assays demonstrated that three of these altered the binding of proteins to the respective *BRCA1* or *BRCA2* promoter regions, including NFYA binding to *BRCA1*:c.‐287C>T and PAX5 binding to *BRCA2*:c.‐296C>T. Clinical classification of variants affecting promoter activity, using existing prediction models, found no evidence to suggest that these variants confer a high risk of disease. Further studies are required to determine if such variation may be associated with a moderate or low risk of BC.

## 1 INTRODUCTION

Genetic susceptibility to breast cancer (BC) is complex. Multiple germline variants have been identified over the past 25 years that are broadly categorized as high, moderate, and low risk. High‐risk variants are generally rare, have a major deleterious effect on gene function, are sufficient to confer a high risk of disease, and are highly penetrant within a family. Nonsense, splicing, large deletions, and some missense changes in *BRCA1* and *BRCA2* fall into this category (reviewed in Walsh et al., 2006). There is also evidence that some alleles confer a moderate risk of cancer. These can include hypomorphic variants in known “high‐risk” cancer syndrome genes (Shimelis et al., 2017; Spurdle et al., 2012), or clear loss‐of‐function alleles in other genes such as *CHEK2*, *PALB2*, and *ATM* (Couch et al., 2017). Low‐risk variants, largely identified by genome‐wide association studies, are usually common and cause subtle functional effects, such as small but significant changes in gene expression due to altered activity of proximal and distal regulatory elements (reviewed in Bogdanova, Helbig, & Dork, 2013; Ghoussaini, Pharoah, & Easton, 2013; Skol, Sasaki, & Onel, 2016). Evidence suggests that combinations of low, moderate, and high‐risk variants could confer a clinically significant risk of disease (Ding et al., 2012; Kuchenbaecker et al., 2017; Sawyer et al., 2012). Identification and evaluation of all such variants is therefore crucial for accurately predicting BC risk.

Use of next generation sequence analysis for germline clinical testing of cancer cases is identifying an increasing number of variants in noncoding regions of cancer susceptibility genes, including promoters, untranslated regions (UTRs), and introns. There are currently no firm recommendations for assessing the relevance of noncoding region variants to clinical testing of Mendelian disease genes, and so the vast majority of such variants are deemed of uncertain clinical significance. This adds to the clinical challenge presented by variants of uncertain significance, namely that they complicate test reporting and genetic counseling, limit patient eligibility for intensive surveillance and gene‐targeted therapies, and prevent gene testing and guided management of relatives (reviewed in Amendola et al., 2015; Eccles et al., 2013; Plon et al., 2011). It is therefore essential that the functional and clinical significance of variants mapping to noncoding regions of the genome is determined.

Gene expression is controlled at many levels with key regulatory elements being housed in noncoding regions of the genome, such as gene promoters, introns, long‐range elements, and 5′ and 3′ UTRs. The *BRCA1* gene is regulated at the transcriptional and posttranscriptional levels, with functional proximal and distal regulatory elements being described in the promoter, introns, and UTRs, by us and others (Brewster et al., 2012; Brown et al., 2002; Santana dos Santos et al., 2017; Saunus et al., 2008; Tan‐Wong, French, Proudfoot, & Brown, 2008; Wardrop, Brown, & kConFab, 2005; Wiedemeyer, Beach, & Karlan, 2014). Although less studied, the *BRCA2* promoter has also been mapped and characterized (reviewed in Wiedemeyer et al., 2014).

Common and rare variations in regulatory elements upstream of genes have been shown to alter gene expression and be associated with disease risk (reviewed in Betts, French, Brown, & Edwards, 2013; Diederichs et al., 2016; Millot et al., 2012). We and others have described germline cancer‐associated variants in the regulatory regions, including large deletions in the *BRCA1* promoter (Brown et al., 2002), and single nucleotide variants in the promoter and/or 5′ UTR of *BRCA1* and *BRCA2* (Evans et al., 2018; Santana dos Santos et al., 2017), *MLH1* promoter (Hitchins et al., 2011), *POLG* promoter (Popanda et al., 2013), *PTEN* promoter (Heikkinen et al., 2011), *TERT* promoter (Horn et al., 2013), *KLHDC7A* and *PIDD1* promoters (Michailidou et al., 2017), *BRCA1* 3′ UTR (Brewster et al., 2012), and BC‐associated Single Nucleotide Polymorphisms (SNPs) in long‐range enhancers of *CCND1* (French et al., 2013).

Cancer risk‐associated variants within regulatory regions are anticipated to mediate an effect on trans‐acting regulatory factors (e.g., transcription factors [TFs] and miRNAs), by disrupting binding of regulatory factors and interactions between regulatory elements, such as promoter–enhancer interactions. For example, a variant in a *Cyclin D1* transcriptional enhancer has been associated with altered binding of the ELK4 TF (French et al., 2013) and a variant within the *BRCA1* 3′UTR has been shown to introduce a functional mir‐103 binding site (Brewster et al., 2012). In addition, a dominantly inherited 5′ UTR *BRCA1* variant was recently shown to be associated with *BRCA1* promoter hypermethylation, which is known to impact TF binding, and associated allelic loss of *BRCA1* expression in two families affected by breast and ovarian cancers (Evans et al., 2018).

In this paper, we describe 141 germline variants in the *BRCA1* and *BRCA2* promoter, identified by members of the ENIGMA consortium in early onset or familial BC patients with no known pathogenic variants in the coding region of these genes. Using a combination of bioinformatic and experimental analyses, we have prioritized and analyzed a subset of variants that are most likely to affect the regulation of *BRCA1* and *BRCA2* and thus have the most potential to contribute to BC risk. TF binding site affinity changes resulting from these variants were subsequently analyzed by information theory (IT)‐based analyses. In parallel, we have assessed if these variants exhibited the features expected for a high‐risk pathogenic *BRCA1* or *BRCA2* variant, on the basis of available clinical and population data.

## 2 MATERIALS AND METHODS

### 2.1 Study design

**Figure 1:**
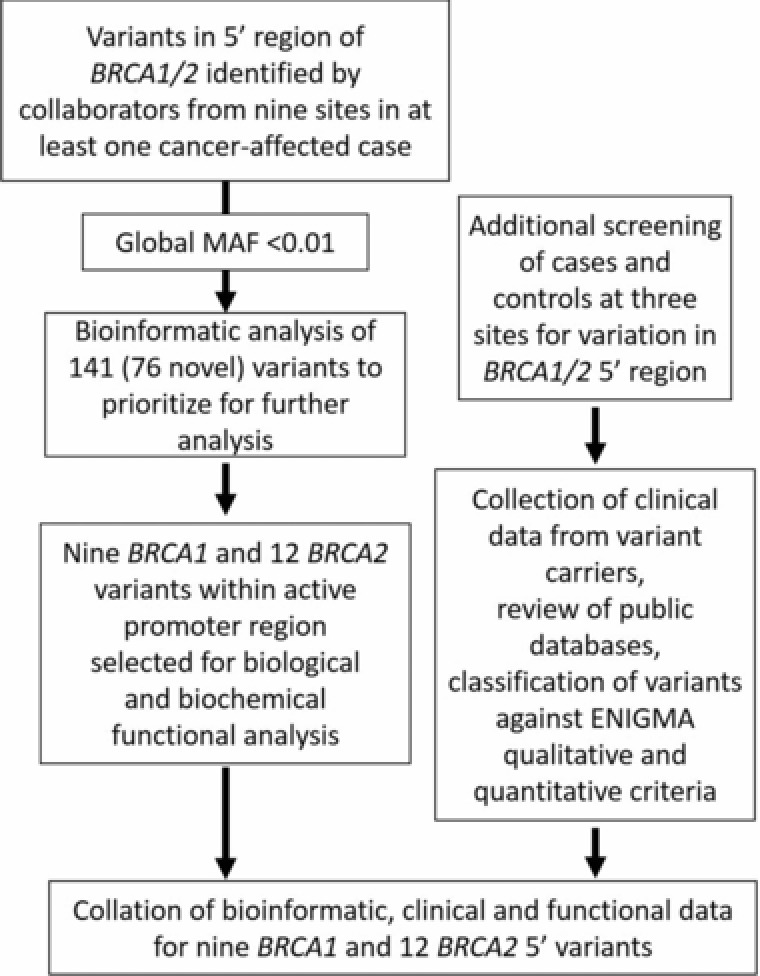
Overview of study design. Outline of the workflow of variant collection, prioritization and analysis

An overview of the study design is shown in Figure 1. Collection of variants at all sites enabled an initial catalogue of variants from which variants were prioritized for functional analysis. Additional screening was carried out at three sites, Maastricht (M), Santiago (S), and Prague (Pr), that included additional patients (M, S, and Pr) and controls (Pr) that expanded the list of variants (Pr), the number of patients (M, S, and Pr), and included control subjects (Pr).

### 2.2 Clinical and control samples

Clinical and genetic data were collected and analyzed in accordance with local human ethics guidelines of the institutions contributing to this study. All participating individuals provided informed consent for their data to be used for research purposes. An overview of the samples analyzed is shown in Table 1. Clinical samples were collected from nine European sites and were originally selected for *BRCA1* and *BRCA2* testing using ascertainment criteria that included family history and young age of BC diagnosis. Female patients who did not carry a pathogenic variant in *BRCA1* or *BRCA2* coding regions or splice junctions were selected for testing of variation in the *BRCA1* and *BRCA2* 5′ regions. The controls were as follows: 661 healthy female individuals recruited through the Immunohematology and Transfusion Medicine Service of INT and Associazione Volontari Italiani Sangue (AVIS) of Milan; 312 healthy females above 60 years of age and with no malignancy in the first filial generation recruited through First Faculty of Medicine, Charles University in Prague (Lhota et al., 2016; Soukupova, Zemankova, Kleiblova, Janatova, & Kleibl, 2016); and 130 healthy females without cancer diagnosis recruited in Santiago de Compostela.

**Table 1:** Samples used in this study

Location	Institution	Samples	Gene region
Paris	Institut Curie, Saint Cloud	686 cases	*BRCA1* 5′region, *BRCA2* 5′region
Milan	IFOM, Fondazione Instituto FIRC di Oncologia Molecolare	772 cases 661 controls	*BRCA1* 5′region
Pisa	Department of Translational Research and New Technologies in Medicine, University of Pisa	80 cases	*BRCA1* 5′region, *BRCA2* 5′region
Santiago de Compostela	Fundación Pública Galega de Medicina Xenómica‐SERGAS, Grupo de Medicina Xenómica‐USC, CIBERER, IDIS	270 cases 130 controls	*BRCA1* 5′region, *BRCA2* 5′region
Copenhagen	Center for Genomic Medicine	1157 cases	*BRCA1* 5′region, *BRCA2* 5′region
Ghent	Center for Medical Genetics, Ghent University Hospital	357 cases	*BRCA1* 5′region, *BRCA2* 5′region
Barcelona	Vall d'Hebron Institute of Oncology	192 cases	*BRCA1* 5′region, *BRCA2* 5′region
Prague	CZECANCA – CZEch CAncer panel for Clinical Aplication, Institute of Biochemistry and Experimental Oncology	2961 cases 312 controls	*BRCA1* 5′region, *BRCA2* 5′region
Maastricht	Department of Clinical Genetics, Maastricht University Medical Centre	900 cases	*BRCA2* 5′region

### 2.3 Identification of variants

Regions containing the *BRCA1* and *BRCA2* promoter and 5′ UTR were sequenced using a range of standard DNA sequencing technologies, and bioinformatic filtering pipelines. Variants mapping to the 2,400 bp region (hg19; chr17:41,278,514 – 41,276,114) of *BRCA1* and the 2,000 bp region (hg19; chr13: 32,888,597‐32,890,597) of *BRCA2* were considered for further analysis. The identified variants in *BRCA1* and *BRCA2* 5′ noncoding regions are numbered whereby the first translated nucleotide of the translation initiation codon is +1 (https://varnomen.hgvs.org/) using the Mutalyzer website (https://mutalyzer.nl/). *BRCA1* is described using NC_000017.10 (hg19 genomic sequence) and NM_007294.3 (transcript). *BRCA2* is described using NC_000013.10 (hg19 genomic sequence) and NM_000059.3 (transcript).

### 2.4 Bioinformatic analysis of variants

As an initial screen, each variant submitted for study was assessed for population frequency using intersection of the variants with dbSNP (version 138 or 150, as the study progressed) within the UCSC Genome browser and Variant Effect Predictor at ENSEMBL (https://www.ensembl.org/info/docs/tools/vep/index.html). Variants with a global minor allele frequency (MAF) of < 0.01 were included in subsequent bioinformatic analyses. Further details of bioinformatics analyses to map active regulatory elements and prioritize variants for functional assays are contained in Supporting Information Methods. Variants were considered to be high priority for experimental analysis if they contained all of the following features: (1) resided in DNaseI or formaldehyde‐assisted isolation of regulatory elements (FAIRE) peaks, (2) coincided with high scores for DNaseI (Base Overlap Signal > 40) or FAIRE (Base Overlap Signal > 10) in a breast cell line, (3) resided in a region of breast cell specific TF binding, (4) overlapped with a TF consensus motif, and (5) were within an evolutionarily conserved element with a high Phastcons score (>0.75). Medium priority variants lacked one or two of these features, whereas low priority variants had only one or none of these features.

#### 2.4.1 *In silico* TF binding analysis

All rare variants were analyzed *in silico* using an IT‐based method (Caminsky et al., 2016; Mucaki et al., 2016) and a modified version of the Shannon pipeline utilizing TF information models built from ENCODE ChIP‐seq datasets (Lu, Mucaki, & Rogan, 2017) to assess potential effects of variants on TF binding. Details of analyses are contained in Supporting Information Methods.

### 2.5 Experimental analysis of variants

#### 2.5.1 Promoter reporter assays

The 499 bp *BRCA1* (chr17:41,277,787‐41,277,289) and 750 bp *BRCA2* (chr13:32,889,230‐32,889,979) promoter regions were cloned into pCR‐Blunt vector (Thermo Fisher, Waltham, MA). Site‐directed mutagenesis was used to introduce variants using the primers listed in Supporting Information Table S1. Plasmids were purified using the QIAprep miniprep kit (Qiagen, Hilden, Germany) as per the manufacturer's instructions. Plasmid preparations were validated using restriction digest and DNA sequencing and inserts were shuttled into pGL3‐Basic luciferase reporter vector (Promega, Madison, WI). All plasmids for transfection were analyzed for DNA conformation on a 1% w/v agarose gel and only plasmids possessing a supercoiled conformation were used for transfections. Transfection details are described in Supporting Information Methods.

The luciferase‐based reporter assay was performed as described previously (Brewster et al., 2012). Positive controls were B1‐Ets, *BRCA1*:c.‐330_‐329delinsTT, that decreases *BRCA1* promoter activity in MCF7 cells (Atlas, Stramwasser, Whiskin, & Mueller, 2000) and B2‐Ets (E2Fmut1: *BRCA2*:c.‐282_‐281delinsAA), that has been shown to decrease *BRCA2* promoter activity in MCF7 cells (Davis, Miron, Andersen, Iglehart, & Marks, 1999). Statistical analyses were performed in GraphPad Prism using one‐way analysis of variance followed by Tukey's post hoc test and values *P* < 0.05 were deemed statistically significant.

#### 2.5.2 Electrophoretic mobility shift assays

Nuclear proteins were extracted as described in Supporting Information Methods and electrophoretic mobility shift assays (EMSAs) were carried out using a Pierce LightShift Chemiluminescent EMSA Kit (Thermo Fisher, Waltham, MA) with modifications described in Supporting Information Methods. For competition and supershift studies, nuclear extracts were initially incubated with unlabeled double‐stranded (ds) competitor probes or antibodies in binding buffer before addition of the biotinylated probe and incubation at room temperature. Positive controls for *BRCA1* and *BRCA2* DNA binding were sequences surrounding the B1‐Ets and B2‐Ets mutations described above.

### 2.6 Qualitative and quantitative classification of variants

Variants were classified according to the ENIGMA classification criteria for variation in *BRCA1* and *BRCA2* (https://enigmaconsortium.org/) to determine whether any of the prioritized variants were associated with a high risk of disease. See Supporting Information Methods for further details.

## 3 RESULTS

### 3.1 Identification and prioritization of sequence variants in *BRCA1* and *BRCA2* 5´ noncoding regions

The 5′ noncoding regions of *BRCA1* and *BRCA2* in early onset or familial BC patients with no known *BRCA1* or *BRCA2* germline pathogenic variant were sequenced at nine different sites as part of an approved ENIGMA (https://enigmaconsortium.org/) project. For the *BRCA1* 5′ region, 6,475 patients were sequenced at eight different sites along with 1,103 controls. For the *BRCA2* 5′ region, 6,603 patients were sequenced at eight different sites as well as 442 controls.

**Figure 2:**
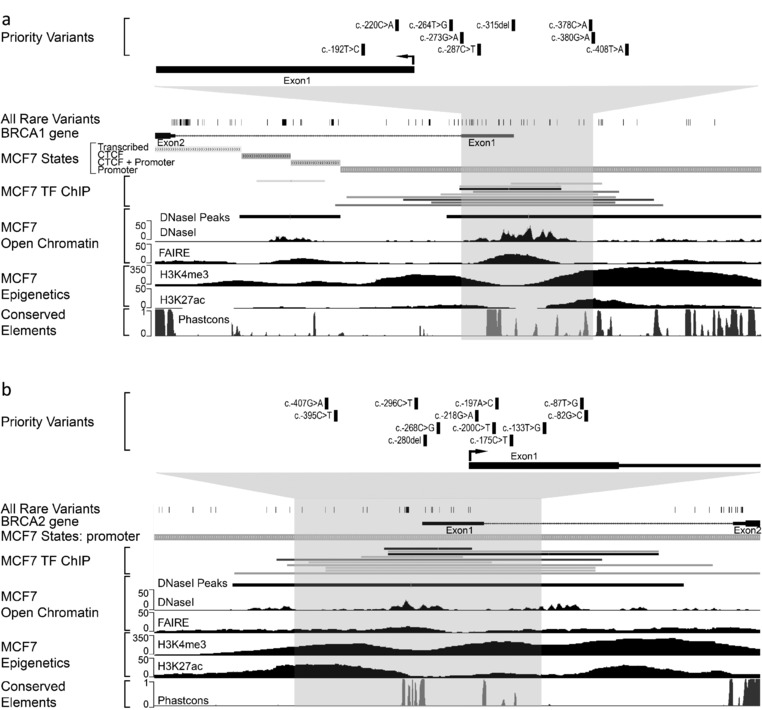
Variants identified in the 5′ regions of *BRCA1* and *BRCA2* map to predicted regulatory elements. Snapshots of the UCSC genome browser showing regions of *BRCA1* (a) and *BRCA2* (b) analyzed by targeted sequencing with available ENCODE regulatory marks derived from MCF7 cells. Chromatin segregation states from regulatory region annotation are shown (MCF7 states). The *BRCA1* and *BRCA2* genomic regions used for functional analyses are highlighted in grey. Prioritized variants within these regions are indicated

After excluding variants with global MAF > 0.01 at time of variant identification, a total of 141 unique single nucleotide variants and short insertions/deletions were identified, 81 in *BRCA1* and 60 in *BRCA2* (Supporting Information Tables S2 and S3). Theses variants have been submitted to the LOVD databases, http://www.lovd.nl/BRCA1 and http://www.lovd.nl/BRCA2. To evaluate the potential of these rare variants to impact gene regulation, we initially undertook a comprehensive bioinformatic analysis. Promoter regions of *BRCA1* and *BRCA2* were defined by bioinformatic predictors including chromatin marks (Figure 2). These regions show the characteristic histone H3 epigenetic marks, including H3K4me3, H3K27ac, and H3K9ac, as well as occupancy by multiple TFs. Of the variants identified in cases only, 22 *BRCA1* and 23 *BRCA2* variants resided within the minimal promoter regions.

To predict the potential impact of variants on promoter activity, we prioritized variants using breast cell specific data for chromatin accessibility and TF occupancy along with evolutionary conservation. Due to the limited breast cell specific TF ChIP‐seq data, we also included ENCODE TF ChIP‐seq and TF consensus motif data from all cell lines. A total of nine *BRCA1* and 12 *BRCA2* variants were selected for further functional analysis (Figure 2; Tables 2 and 3).

**Table 2:** *BRCA1* prioritized variants

Gene	hg19 position (chr17)	Variant name^a^	rsID	Global MAF in dbSNP	TF motif (ENCODE)^b^	Bioinformatic priority
*BRCA1*	g.41277676A>T	c.‐408T>A	Novel		CEBPB	High/medium
*BRCA1*	g.41277648C>T	c.‐380G>A	Novel		RXRA	High/medium
*BRCA1*	g.41277646G>T	c.‐378C>A	rs186775935	0.00040	RXRA	High/medium
*BRCA1*	g.41277583del	c.‐315del	rs901029407	0.00003	ATF1,2,3, CREB1^c^	Medium
*BRCA1*	g.41277555G>A	c.‐287C>T	Novel		NFYA, NFYB	High/medium
*BRCA1*	g.41277541C>T	c.‐273G>A	rs112960339	0.00499		Medium
*BRCA1*	g.41277532A>C	c.‐264T>G	rs904148166	0.00003		Medium
*BRCA1*	g.41277488G>T	c.‐220C>A	Novel			Medium
*BRCA1*	g.41277460A>G	c.‐192T>C	rs113323025	0.00519		Medium

TF, transcription factors.

a Based on NM_007294.3.

b Overlap with TF motif in ENCODE TF‐ChIP datasets from all cells.

c Variant overlaps this motif, but the deletion does not alter the motif sequence.

**Table 3:** *BRCA2* prioritized variants

Gene	hg19 Position (Chr13)	Variant name^a^	rsID	Global MAF in dbSNP	TF motif (ENCODE)^b^	Bioinformatic priority
*BRCA2*	g.32889437G>A	c.‐407G>A	rs36221751	0.0018		Medium
*BRCA2*	g.32889449C>T	c.‐395C>T	Novel			Medium
*BRCA2*	g.32889548C>T	c.‐296C>T	rs563971900	0.0004	PAX5	High/medium
*BRCA2*	g.32889564delG	c.‐280del	Novel		ELF1, GABPA, ELK1,4	High
*BRCA2*	g.32889576C>G	c.‐268C>G	Novel			High/medium
*BRCA2*	g.32889626G>A	c.‐218G>A	Novel			Medium
*BRCA2*	g.32889644C>T	c.‐200C>T	Novel		MAZ	Medium
*BRCA2*	g.32889647A>C	c.‐197A>C	rs370721506	NA	MAZ	Medium
*BRCA2*	g.32889669C>T	c.‐175C>T	rs55880202	0.0058		Medium
*BRCA2*	g.32889711T>G	c.‐133T>G	Novel			Medium
*BRCA2*	g.32889757T>G	c.‐87T>G	Novel			Medium/low
*BRCA2*	g.32889762G>C	c.‐82G>C	Novel			Medium/low

NA, no data available, TF, transcription factors.

a Based on NM_000059.3.

b Overlap with TF motif in ENCODE TF‐ChIP datasets from all cells.

### 3.2 *BRCA1* and *BRCA2* promoter activity is altered by 5′ noncoding sequence variants

**Figure 3:**
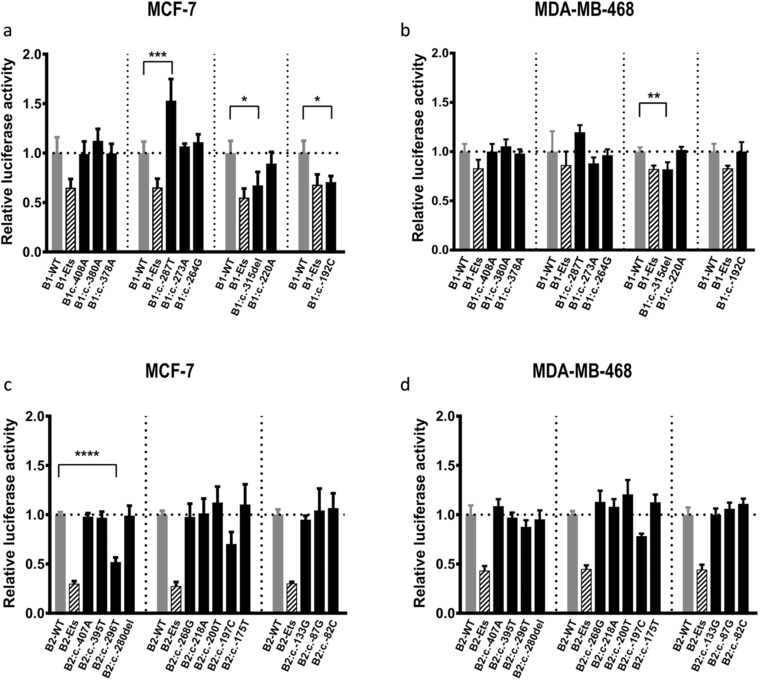
Variants mapping to the 5′ regions of *BRCA1* and *BRCA2* alter promoter activity in MCF7 and MDA‐MB‐468 breast cancer cells. MCF7 (a and c) and MDA‐MB‐468 cells (b and d) were transfected with pGL3 vectors where luciferase expression is controlled by a portion of the *BRCA1* (B1) (a and b) or *BRCA2* (B2) (c and d) promoter. Cells were transfected with plasmids containing the wild‐type (WT) promoter sequence (grey bars), positive control (B1‐Ets or B2‐Ets; striped bars) or the indicated variants (black bars). Luciferase expression was normalized to a cotransfected pRL‐TK plasmid. Data represent the average of three independent biological replicates ± standard deviation (*SD*). The horizontal dotted line represents WT promoter activity set at 1.0‐fold. The vertical dotted lines demarcate individual experiments that include WT, positive control, and variant containing plasmids. (* *P* ˂ 0.05; ** *P* ˂ 0.01, *** *P* ˂ 0.005, **** *P* ˂ 0.0001)

To examine the potential effect of the 21 prioritized *BRCA1* and *BRCA2* 5′ noncoding variants on regulatory activity, promoter activity was measured using luciferase assays in MCF7 and MDA‐MB‐468 BC cell lines. Two of the nine prioritized *BRCA1* variants decreased *BRCA1* promoter activity relative to the wild‐type (WT) construct (Figure 3a and 3b). *BRCA1*:c.‐315del significantly decreased the *BRCA1* promoter luciferase activity in both cell lines, whereas *BRCA1*:c.‐192C decreased luciferase activity in the MCF7 cell line. Furthermore, one variant, *BRCA1*:c.‐287T, displayed increased activity relative to the WT construct in the MCF7 cell line. For *BRCA2*, one of the 12 variants, *BRCA2*:c.‐296T, decreased *BRCA2* promoter activity relative to the WT construct in the MCF7 cell line (Figure 3c and 3d).

### 3.3 *In silico* analyses of *BRCA1* and *BRCA2* 5′ variants predict alterations in TF binding

*BRCA1* and *BRCA2* promoters are regulated by a complex array of DNA‐binding proteins and transcriptional coactivators and corepressors (reviewed in McCoy, Mueller, & Roskelley, 2003; Mueller & Roskelley, 2003; Wiedemeyer et al., 2014). In silico analysis was carried out to examine whether the *BRCA1* and *BRCA2* promoter variants shown to alter luciferase activity (see above) are likely to affect binding of trans‐acting protein factors in breast cells.

Interrogation of ENCODE ChIP‐seq datasets derived from breast cell lines show that, although the number of datasets is limited, TFs bind to regions encompassing the prioritized variants (Figure 2 and Supporting Information Figure S1). ENCODE ChIP‐seq data from other cell lines indicate that some variants are located within consensus motifs for specific TFs associated with these regions (Tables 2 and 3; Supporting Information Figure S1). *BRCA1:c*.‐287C>T overlaps with the consensus binding motif for CCAAT Box binding factors and *BRCA2:c*.‐296C>T is located within the consensus motif for PAX5.

IT analysis of the prioritized variants showed that the binding strengths of several TFs are predicted to be altered by the *BRCA1* and *BRCA2* variants (Table 4 and Supporting Information Table S4). All of the variants that altered promoter activity were predicted to have consequences on TF binding. *BRCA1*:c.‐287C>T and *BRCA2*:c.‐296C>T are predicted to disrupt binding of CCAAT Box binding factors and PAX5, respectively. *BRCA1*:c.‐315del is predicted to disrupt the binding of TCF7L2 but creates a POU2F2 (also known as Oct‐2) binding site. *BRCA1*:c.‐192T>C is predicted to strengthen a RFX5 site and creates an ETS1 site.

**Table 4:** Information theory analysis of prioritized *BRCA1/2* variants

Variant name	TF motif (ENCODE)	Consequences
*BRCA1*:c.‐408T>A	CEBPB	CEBPB site weakened (did not meet stringent filtering thresholds)
*BRCA1*:c.‐380G>A	RXRA	Weak RXRA and IRF3 sites weakened, HNF4G site weakened.
*BRCA1*:c.‐378C>A	RXRA	RXR unchanged, HSF1 site lost and GR site created
*BRCA1*:c.‐315del	ATF1,2,3, CREB1^a^	TCF7L2 site lost and POU2F2 created
*BRCA1*:c.‐287C>T	NFYA, NFYB	NFYA and NFYB sites lost, weak PBX3 site created
*BRCA1*:c.‐273G>A		Altered TF strength did not fulfill stringent filtering thresholds^b^
*BRCA1*:c.‐264T>G		BHLHE32 and MYC sites created.
*BRCA1*:c.‐220C>A		Altered TF strength did not fulfill stringent filtering thresholds^b^
*BRCA1*:c.‐192T>C		ETS1 site created, weak RFX5 site strengthened.
*BRCA2*:c.‐407G>A		Weak MEF2A site strengthened, GATA2 site lost.
*BRCA2*:c.‐395C>T		TEAD4 site lost.
*BRCA2*:c.‐296C>T	PAX5	PAX5 site weakened.
*BRCA2*:c.‐280del	ELF1, GABPA, ELK1,4	GABPA site unchanged, MXI1 andTCF3 sites lost.
*BRCA2*:c.‐268C>G		Altered TF strength did not meet filtering thresholds^b^
*BRCA2*:c.‐218G>A		Altered TF strength did not meet filtering thresholds^b^
*BRCA2*:c.‐200C>T	MAZ^c^	KLF1 site abolished.
*BRCA2*:c.‐197A>C	MAZ^c^	SP4 weakened, GR site weakened, TCF3 site created
*BRCA2*:c.‐175C>T		Altered TF strength did not fulfill stringent filtering thresholds^b^
*BRCA2*:c.‐133T>G		Altered TF strength did not fulfill stringent filtering thresholds^b^
*BRCA2*:c.‐87T>G		Altered TF strength did not fulfill stringent filtering thresholds^b^
*BRCA2*:c.‐82G>C		Altered TF strength did not fulfill stringent filtering thresholds^b^

a Variant overlaps this motif, but the deletion does not alter the motif sequence.

b Change in information did not fulfill stringent filtering criteria, where [A] site *R*_i_ < *R*_sequence_–1 standard deviation of TF model, or [B] where Δ*R*_i_ < 4 bits.

c No MAZ binding model available.

### 3.4 5′ variants in *BRCA1* and *BRCA2* alter protein–DNA interactions in EMSA analyses

**Figure 4:**
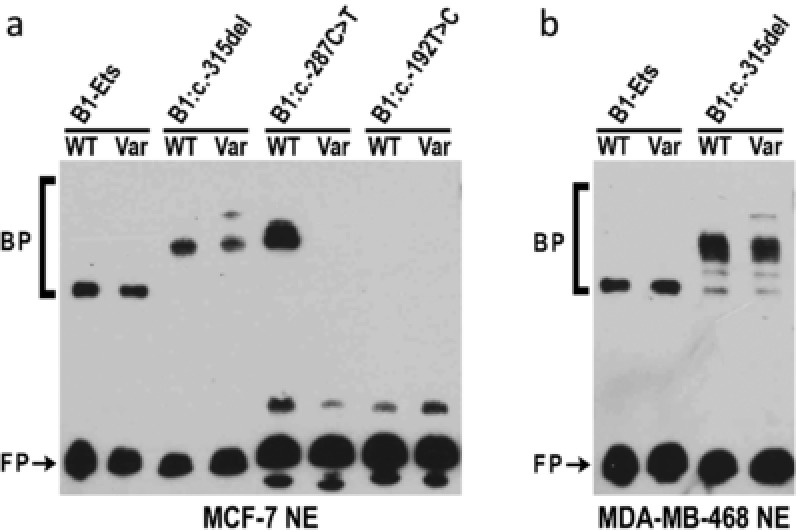
Variants in the 5′ regions of *BRCA1* alter DNA:protein complex formation. Electrophoretic mobility shift assay (EMSA) reactions were performed with 3′ biotinylated double‐stranded DNA probes from the *BRCA1* 5′ region and nuclear extracts (NE) from (a) MCF7 or (b) MDA‐MB‐468 cells. DNA probes contained either wild‐type (WT) or variant (Var) sequences. Free unbound probe (FP) and probe bound by nuclear proteins (BP) are indicated

To examine potential alterations in the binding of nuclear proteins from breast cells by the *BRCA1* and *BRCA2* promoter variants that altered luciferase activity, we carried out EMSA analysis. For *BRCA1*, two of three analyzed variants, c.‐315del and c.‐287C>T, displayed allele‐specific protein binding (Figure 4). For probes containing the region surrounding the *BRCA1*:c.‐315del variant, changing the WT sequence to the variant sequence resulted in the enhanced binding of a slower migrating band (Figure 4a and 4b). For probes containing the region surrounding the *BRCA1*:c.‐287C˃T variant, introduction of the variant sequence resulted in almost complete loss of protein binding to the probe (Figure 4a).

**Figure 5:**
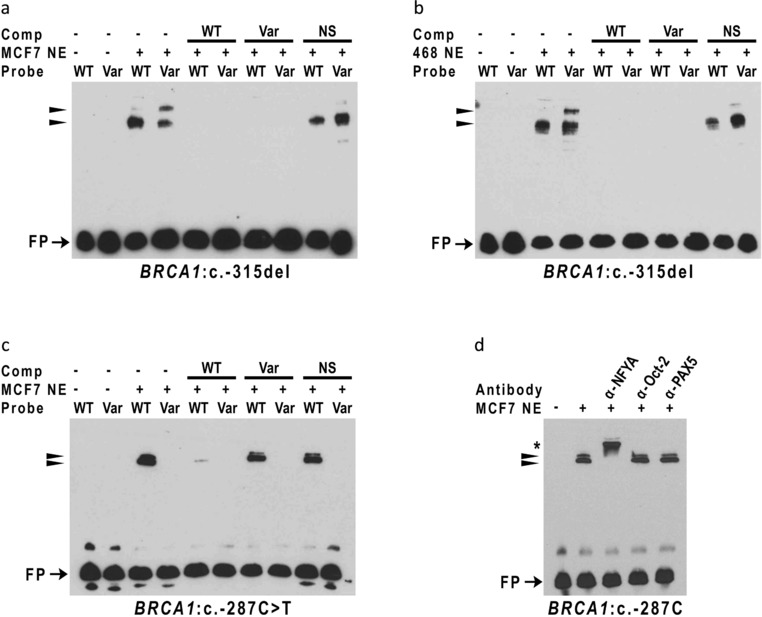
Variant sequences in the *BRCA1* 5′ region alter specific DNA:protein complex formation. Competition electrophoretic mobility shift assay (EMSAs) were performed using 3′ biotinylated double‐stranded DNA probes containing sequences from the *BRCA1* 5′ region surrounding the B1:c.‐315del (a and b) and B1:c.‐287C>T (c) variants. DNA probes containing the wild‐type (WT) or variant (Var) sequence were incubated with nuclear extracts from MCF7 cells (MCF7 NE) or MDA‐MB 468 cells (468 NE) in the presence (+) or absence (–) of unlabeled WT, Var, or nonspecific (NS) competitor (Comp) DNA. Free unbound probe (FP) and specific DNA:protein complexes (arrowheads) are indicated. Supershift experiments (d) were performed with the *BRCA1*:c.‐287C (WT) probe and antibodies to NFYA, Oct‐2 (POU2F2) and PAX5. The supershifted NFYA complex is indicated by asterisk (*)

To determine if the DNA‐protein interactions were specific, competition experiments were performed. In the case of *BRCA1*:c.‐315del, all bands were competed by both the WT and the variant containing probes in two cell lines (Figure 5a and 5b). For *BRCA1*:c.‐287C>T, only the WT probe was able to compete for binding (Figure 5c). The nonspecific probe from an unrelated region of the *BRCA1* promoter did not compete any bands showing that the bands seen in the EMSA were specific.

**Figure 6:**
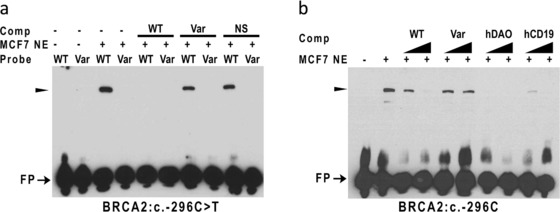
Variants in the 5′ region of *BRCA2* alter specific DNA:protein complex formation. Competition electrophoretic mobility shift assay (EMSAs; a) were performed using 3′ biotinylated double‐stranded (ds) DNA probes containing sequences from the *BRCA2* 5′ region surrounding the BRCA2:c.‐296C>T variant. DNA probes containing the wild‐type (WT) or variant (Var) sequence were incubated with nuclear extracts from MCF7 cells (MCF7 NE) in the presence (+) or absence (–) of unlabeled WT, Var, or nonspecific (NS) competitor (Comp) DNA. Cross‐competition EMSAs (b) contained *BRCA2* WT sequences and increasing concentrations of ds competitor DNA containing unlabeled WT, Var, or PAX5 binding sites from the *hCD19* gene and D‐amino acid oxidase gene (*hDAO*). Free unbound probe (FP) and specific DNA:protein complexes (arrowheads) are indicated

Analysis of the regions of the *BRCA2* promoter using EMSA revealed that region containing the *BRCA2*:c.‐296C>T variant bound nuclear proteins from MCF7 nuclear extracts and that this interaction was dramatically reduced by introduction of the variant sequence (Figure 6a). Competition experiments showed that these interactions were specific and not competed by a nonspecific probe from an unrelated region of the *BRCA1* promoter (Figure 6a).

To determine the effect of these variants on the binding of specific TFs, competition and supershift analyses were performed. *BRCA1*:c.‐287C>T overlaps with the consensus binding motif for CCAAT Box binding factors, NFYA and NFYB (Table 2 and Supporting Information Figure S1a), and IT analysis predicts that the variant disrupts binding of these TFs (Table 4). Consistent with these predictions, supershift experiments show that *BRCA1*:c.‐287C>T disrupts binding of NFYA to this region (Figure. 5d). In addition, we analyzed *BRCA2*:c.‐296C>T, which maps within the consensus binding motif for PAX5 (Table 2 and Supporting Information Figure S1b), and is predicted by IT analysis to disrupt binding of PAX5 (Table 4), by cross‐competition experiments using known PAX5 binding sites from *hCD19* (Kozmik, Wang, Dorfler, Adams, & Busslinger, 1992) and *hDAO* (Tran et al., 2015) genes. These experiments show that known PAX5 binding sites compete efficiently for binding of nuclear proteins to the *BRCA2* promoter region, indicating that PAX5 binding is reduced as a consequence of the nucleotide sequence change (Figure. 6b). In contrast, supershift experiments for POU2F2 (Oct‐2) showed no evidence for *BRCA1*:c.‐315del causing a change in binding of POU2F2 in the cell line used (data not shown).

### 3.5 Clinical classification of *BRCA1* and *BRCA2* 5′ noncoding sequence variants

Variants were classified according to the ENIGMA guidelines, which are calibrated for classification of variants as high risk, using available population frequency and/or clinical data (Supporting Information Tables S5 and S6). In this context, the term pathogenicity refers to a variant that confers a high risk of disease. Importantly, these classification guidelines do not identify those variants that confer a moderate or low risk of disease.

Of those variants identified in cases only, 26/70 (37%) of BRCA1 variants had been reported in dbSNP at study initiation (maximum global frequency = 0.006; Supporting Information Table S2), and 22/54 (41%) of *BRCA2* variants observed in cases only were identified in dbSNP (maximum global frequency = 0.006; Supporting Information Table S3). Review of variant frequency in public reference groups identified 21 variants that were classifiable, as Not Pathogenic, based on frequency in control groups (Supporting Information Table S5): six *BRCA1* and five *BRCA2* variants were observed at >1% frequency in population subgroups (stand‐alone evidence against pathogenicity, when detected in a nonfounder outbred population group); six *BRCA1* and four *BRCA2* variants occurred at frequency 0.001–0.01 (range 0.0014–0.0076) in at least five individuals in the reference set, which combined with a low assumed prior is considered sufficient as evidence against pathogenicity (Supporting Information Table S5). Frequency data from controls screened for this study also supported the frequency‐based classifications for eight of these 21 variants (Supporting Information Table S5).

Segregation analysis for seven informative families aided classification for six variants, whereas histopathology likelihood ratios (LRs) derived for 24 tumors altered classification for 10 variants (Supporting Information Table S6). Combining findings from qualitative and quantitative methods, most variants (113/141; 80%) remained Class 3 Uncertain, largely due to a lack of data.

A total of 27/141 (19%) variants were classified as Not Pathogenic or Likely Not Pathogenic. Of the 21 variants prioritized for functional analysis, eight variants (38%) were classified as Not Pathogenic or Likely Not Pathogenic based on frequency information and/or multifactorial analysis (Table 5), including two variants (*BRCA1:c*.‐192T>C and *BRCA2:c*.‐296 C > T) that were shown to decrease promoter activity and in the case of *BRCA2:c*.‐296 C>T also resulted in perturbed TF binding. Taken together this analysis indicates that none of the variants shown to affect function in this study are associated with a high risk of disease. This analysis is silent, however, on whether these variants may confer a moderate or low risk of disease.

**Table 5:** Classification of prioritized variants

Gene	Genomic location (hg19)	HGVS c. nomenclature	Luciferase result	Combined interpretation of frequency data & multifactorial analysis	Highest MAF (population, database)	Prior probability of pathogenicity	Segregation Bayes score (# families)	Tumor histopathology likelihood ratio (# tumors)	Combined odds for causality	Posterior probability of pathogenicity^c^
*BRCA1*	g.41277676A>T	c.‐408T>A	No effect	Uncertain		0.02				
*BRCA1*	g.41277648C>T	c.‐380G>A	No effect	Uncertain		0.02		1.67 (1)	1.67	NA
*BRCA1*	g.41277646G>T	c.‐378C>A	No effect	Uncertain	0.0015 (African, 1,000 Genomes)	0.02				
*BRCA1*	g.41277583del	c.‐315del	Decrease	Uncertain		0.02				
*BRCA1*	g.41277555G>A	c.‐287C>T	Increase	Uncertain		0.02		0.64 (1)	0.64	NA
*BRCA1*	g.41277541C>T	c.‐273G>A	No effect	Not pathogenic^a^	0.0159 (African, 1,000 Genomes)	0.02				
*BRCA1*	g.41277532A>C	c.‐264T>G	No effect	Uncertain		0.02		0.51 (1)	0.51	NA
*BRCA1*	g.41277488G>T	c.‐220C>A	No effect	Uncertain		0.02				
*BRCA1*	g.41277460A>G	c.‐192T>C	Decrease	Not pathogenic^a^	0.0159 (African, 1,000 Genomes)	0.02				
*BRCA2*	g.32889437G>A	c.‐407G>A	No effect	Not pathogenic^b^	0.0080 (Prague, this study)	0.02		0.55 (6)	0.55	NA
*BRCA2*	g.32889449C>T	c.‐395C>T	No effect	Uncertain		0.02				
*BRCA2*	g.32889548C>T	c.‐296C>T	Decrease	Not pathogenic^b^	0.0080 (Prague, this study)	0.02	3.07 (1)	1.91 (8)	5.87	0.1069
*BRCA2*	g.32889564delG	c.‐280del	No effect	Uncertain		0.02		0.69 (1)	0.69	NA
*BRCA2*	g.32889576C>G	c.‐268C>G	No effect	Uncertain		0.02				
*BRCA2*	g.32889626G>A	c.‐218G>A	No effect	Likely not pathogenic		0.02	0.52 (1)	0.72 (1)	0.38	0.0076
*BRCA2*	g.32889644C>T	c.‐200C>T	No effect	Likely not pathogenic		0.02		0.37 (1)	0.37	0.0075
*BRCA2*	g.32889647A>C	c.‐197A>C	No effect	Not pathogenic^b^	0.0014 (African, FLOSSIES)	0.02		1.08 (1)	1.08	NA
*BRCA2*	g.32889669C>T	c.‐175C>T	No effect	Not pathogenic^a^	0.0197 (African, FLOSSIES)	0.02				
*BRCA2*	g.32889711T>G	c.‐133T>G	No effect	Uncertain		0.02				
*BRCA2*	g.32889757T>G	c.‐87T>G	No effect	Uncertain		0.02				
*BRCA2*	g.32889762G>C	c.‐82G>C	No effect	Uncertain		0.02				

NA, not applicable: multifactorial classification not assigned as the combined odds of causality were insufficient (≥0.5 and ≤2) to derive a posterior probability of pathogenicity (Vallee et al., 2016).

a Not pathogenic based on frequency > 1% in an outbred sampleset.

b Variant allele assigned a low prior probability of pathogenicity of 0.02 assuming conservatively that 2/100 of such variants might be associated with a high risk of cancer and allele frequency ≥0.001 and < 0.01 in outbred sample set.

c Posterior probabilities used to assign IARC 5‐tier class as described in Plon et al., 2008.

## 4 DISCUSSION

Next generation sequencing and gene panel testing enable rapid analysis of gene regions that have previously not been included in standard screening procedures, including promoters, UTRs, introns, and extragenic regions. It is hypothesized that variants in these regions have potential to modulate gene expression (Stranger et al., 2005; Stranger et al., 2007) and impact on relative disease risk, possibly in collaboration with multiple other low‐, moderate‐, and high‐risk variants (Manolio et al., 2009). This extends and validates our previous study (Santana dos Santos et al., 2017) by using a larger number patients analyzed over nine geographical locations, identifying additional BC‐associated variants, and showing that a subset of these variants modulate binding of specific TFs. Further, we have compared results from our bioinformatics and functional analysis to variant classifications based on ENIGMA *BRCA1/2* guidelines for high‐risk variation in these genes.

Through targeted sequencing of over 6,000 early onset/familial BC patients, we identified 141 single nucleotide variants and small indels mapping to the 5′ noncoding regions of BRCA1 and BRCA2. Of these, four (*BRCA1*:c.‐315del, *BRCA1*:c.‐287C>T, *BRCA1*:c.‐192T>C, and *BRCA2*:c.‐296C>T) caused a significant change in promoter activity. The observed alterations in *BRCA1* and *BRCA2* promoter activity are of a similar magnitude to that seen with other germline variants associated with BC risk (Michailidou et al., 2017), including a variant in the *TERT* promoter, which creates a new binding site for Ets factors and results in a 1.2–1.5‐fold increase in luciferase activity in a promoter reporter assay (Horn et al., 2013), and variants in the promoters of *KLHDC7A* and *PIDD1* (Michailidou et al., 2017). Although this supports the hypothesis that moderate change in promoter activity can be associated with disease risk, further work is needed to confirm this.

One of the four variants significantly altered luciferase activity in both tested cell lines, whereas the remaining three variants only affected luciferase activity in MCF7 cells. This may reflect the differential availability of crucial TFs in MDA‐MB‐468 cells (Kao et al., 2009) and highlights the importance of undertaking that assays for functional activity of variants in more than one cell line. Three variants, *BRCA1*:c.‐380G>A, *BRCA2*:c.‐296C>T, and *BRCA2*:c.‐218G>A, were also analyzed in our earlier paper (Santana dos Santos et al., 2017). Although the cell lines used in the two studies were different (MDA‐MB‐231 in Santana dos Santos et al., 2017 and MCF7 and MDA‐MB‐468 here), the trends are the same in five out of six analyses. The difference for *BRCA2*:c.‐296C>T, which causes a significant decrease in MDA‐MB‐231 and MCF7 cells, but not MDA‐MB‐468 cells, may again be indicative of differential gene expression in BC cell lines (Kao et al., 2009). Overall, however, the consistency of results performed in two separate laboratories underscores the robustness of the assay system.

Some variants were associated with a decrease in promoter activity, whereas others were associated with an increase. As TFs can function as activators or repressors, a variant‐associated change in TF binding can result in either a decrease or an increase in promoter (or other regulatory element) activity. Differences in the quanta and direction of promoter activity have been reported previously (e.g., Fraile‐Bethencourt et al., 2018; Santana dos Santos et al., 2017) and have also been shown to differ between cell lines potentially reflecting the availability of TFs or cofactors (e.g., Zn).

Three of the variants, *BRCA1*:c.‐315del, *BRCA1*: c.‐287C>T, and *BRCA2*:c.‐296C>T, altered protein binding. ENCODE ChIP‐seq data from BC cell lines indicate candidate proteins that are bound to the genomic regions containing these variants (Figure 2 and Supporting Information Figure S1). These include E2F1, CEBPB, GATA3, Max, ELF1, GABP, and FOXA1 for *BRCA1* and E2F1, MYC, ELF1, GABP, Max, and PML for *BRCA2*. Interestingly, a number of these factors have previously been implicated in BC.

In addition, ENCODE ChIP‐seq data from cell lines derived from tissues other than breast indicate that the variants that affect protein binding are located within consensus motifs for specific TFs associated with these regions (Tables 2 and 3; Supporting Information Figure S1). *BRCA1*:c.‐287C>T overlaps with the consensus binding motif for CCAAT Box binding factors, *BRCA1*:c.‐315del is located in a consensus motif for CREB/ATF proteins, although the deletion does not modify this motif, and *BRCA2*:c.‐296C>T is located within the consensus motif for PAX5. IT analysis also predicts that all these variants alter TF binding (Table 4 and Supporting Information Table S4). We show that *BRCA1*:c.‐287C>T disrupts the binding of NFYA to the *BRCA1* promoter region. Furthermore, we present evidence that *BRCA2*:c.‐296C>T disrupts the binding of PAX5. *BRCA1*:c.‐315del lies in the so‐called positive regulator region that has been shown to bind GABPα, CREB, and AP‐1 proteins (Atlas et al., 2000; Atlas, Stramwasser, & Mueller, 2001; Graves, Zhou, MacDonald, Mueller, & Roskelley, 2007; Suen & Goss, 1999; Thakur & Croce, 1999). Although these proteins are generally considered activators of transcription, repression of promoter activity by *BRCA1*:c.‐315del suggests the recruitment of an additional transcriptional repressor or corepressor to this region. IT analysis predicts creation of a binding site for POU2F2, a known repressor; however, we found no evidence to suggest that this variant increased POU2F2 binding in the cell line used, although it is possible that changes may be observable in other cell lines. Biochemical studies, including mass spectrometry, will be required to validate and discover other alterations in TF binding.

One variant, *BRCA1*:c.‐287C>T, increased promoter activity and decreased protein:DNA interactions. This increase in promoter activity was unanticipated because this variant is within a consensus motif for the CCAAT box binding proteins, NFYA and NFYB, and mutation of this CCAAT box has previously been shown to reduce *BRCA1* promoter activity in MCF7 cells (Bindra et al., 2005; Xu, Chambers, & Solomon, 1997). This variant also decreases promoter activity in MDA‐MB‐231 cells (Santana dos Santos et al., 2017). Here, we show that the *BRCA1*:c.‐287C>T variant reduces NFYA binding. Importantly, NFY proteins can function as transcriptional activators or repressors depending on recruitment of corepressors or coactivators (Peng & Jahroudi, 2002; Peng et al., 2007) and recruitment of TFs to neighboring sequences (Zhu et al., 2012) indicating possible mechanisms for divergent activities of NFY proteins at this site.

*BRCA1*:c.‐192T>C, which lies in the 5′UTR, decreased reporter activity but did not bind any proteins from MCF7 nuclear extracts in EMSA analysis. Possibly, EMSA binding conditions are not optimal for binding of factors to this sequence or alternatively, this reduction in promoter activity could be by posttranscriptional mechanisms as seen for *BRCA2*:c.‐26G>A (Gochhait et al., 2007).

Using existing prediction models developed for high risk variants, population frequency and clinical information classified 27 variants as "Not Pathogenic" or "likely Not Pathogenic." This included two *BRCA1* and six *BRCA2* variants with functional assay data available, six with no statistically significant effect on promoter activity, and two that decreased promoter activity in vitro. These two variants, *BRCA1:c*.‐192T>C and *BRCA2:c*.‐296C>T, were observed in population subgroup controls; notably *BRCA1:c*.‐192T>C was observed at a frequency of >1%, which is considered stand‐alone evidence against pathogenicity (defined as high risk of cancer) for *BRCA1/2* variation. This suggests that promoter region variants, irrespective of bioinformatic prediction or functional assay results, are unlikely to be associated with a high risk of cancer. This is consistent with current evidence from ENIGMA studies (de la Hoya et al., 2016), which suggest that an allele resulting in only ∼20–30% expression of *BRCA1* transcript/s encoding functional transcripts is not associated with high risk of BC. The low impact of these variants on risk is likely to reflect the complex interplay of TFs and DNA elements, and possible redundancy in the system. For example, a variant in one TF binding site within a cluster may be buffered by other binding sites and thus insufficient on its own to reduce gene expression markedly (Lu & Rogan, 2018).

Given that moderate‐ and low‐risk variants often occur in >1% of the population, and that the remaining 13 variants had insufficient evidence available to assess clinical significance, we cannot exclude the possibility that *BRCA1/2* promoter region variants, in particular those with proven functional effect, may be associated with a moderate or low risk of cancer. This indicates an urgent need to further develop prediction models to accommodate criteria for moderate‐ or low‐risk variants by extending the *BRCA1/2*‐specific criteria developed by ENIGMA (https://www.enigmaconsortium.org/), or even the generic variant classification criteria developed by the American College of Medical Genetics for Mendelian disorders (Richards et al., 2015).

This study has evaluated the significance of single nucleotide variants and small indels mapping to the 5′ noncoding region of *BRCA1* and *BRCA2* using bioinformatic, biological, and biochemical analyses in combination with consideration of clinical data that inform qualitative and quantitative variant classification. We present data to suggest that a subset of these variants have functional effects on gene regulation. We also present evidence that variants mapping to and affecting the function of *BRCA* promoters are not likely to be associated with a high risk of cancer. We propose that studies of differing design, such as very large‐scale case‐control sequencing studies able to detect rare variation, will be required to address if a low to moderate risk of cancer may be associated with *BRCA1/2* regulatory region variation that has not been captured to date by genome‐wide association genotyping platforms. We believe that the bioinformatic and functional analysis presented will be important to define the design and interpretation of such future sequencing studies. We also believe that this study highlights the challenges associated with classifying variants with respect to low or moderate disease risk, and the need to be cautious in the clinical use of information on individual variants that is likely to be one of many factors contributing to disease risk.

## Supporting information

Supplementary Figure S1. Variants in *BRCA1* and *BRCA2* overlap with potential TF binding sites. Snapshots of the UCSC genome browser showing *BRCA1* (A) and *BRCA2* (B) prioritized variants and ENCODE ChIP‐seq data from multiple cell lines and available breast cell specific TF ChIPseq data. TF consensus motifs within the ENCODE ChIP‐seq dataset tracks are displayed in green. Genomic position of variants that alter luciferase activity are indicated by vertical lines.
Supplementary Table S1. Oligonucleotides used in this study
Supplementary Table S2. Overview of rare variants in the *BRCA1* 5'upstream region in patients and controls.*
Supplementary Table S3. Overview of rare variants in the *BRCA2* 5'upstream region in patients and controls.*
Supplementary Table S4. Information Theory Analysis of Prioritized Variants
Supplementary Table S5. Clinical classification of *BRCA1* and *BRCA2* 5' noncoding variants
Supplementary Table S6. Tumour histopathology status for *BRCA1* and *BRCA2* 5' noncoding variants

Supplementary methods
