# Supplementary material for: BRCA1 and BRCA2 5′ noncoding region variants identified in breast cancer patients alter promoter activity and protein binding
Source: Hum Mutat. 2018 Sep 24;39(12):2025–39. doi: 10.1002/humu.23652 (PMC6282814; doi:10.1002/humu.23652)
Supplement: Supplementary file 1 — Supplementary Figure S1. Variants in BRCA1 and BRCA2 overlap with potential TF binding sites. Snapshots of the UCSC genome browser showing BRCA1 (A) and BRCA2 (B) prioritized variants and ENCODE ChIP‐seq data from multiple cell lines and available breast cell specific TF ChIPseq data. TF consensus motifs within the ENCODE ChIP‐seq dataset tracks are displayed in green. Genomic position of variants that alter luciferase activity are indicated by vertical lines. Supplementary Table S1. Oligonucleotides used in this study Supplementary Table S2. Overview of rare variants in the BRCA1 5'upstream region in patients and controls.* Supplementary Table S3. Overview of rare variants in the BRCA2 5'upstream region in patients and controls.* Supplementary Table S4. Information Theory Analysis of Prioritized Variants Supplementary Table S5. Clinical classification of BRCA1 and BRCA2 5' noncoding variants Supplementary Table S6. Tumour histopathology status for BRCA1 and BRCA2 5' noncoding variants [file HUMU-39-2025-s001.pdf]

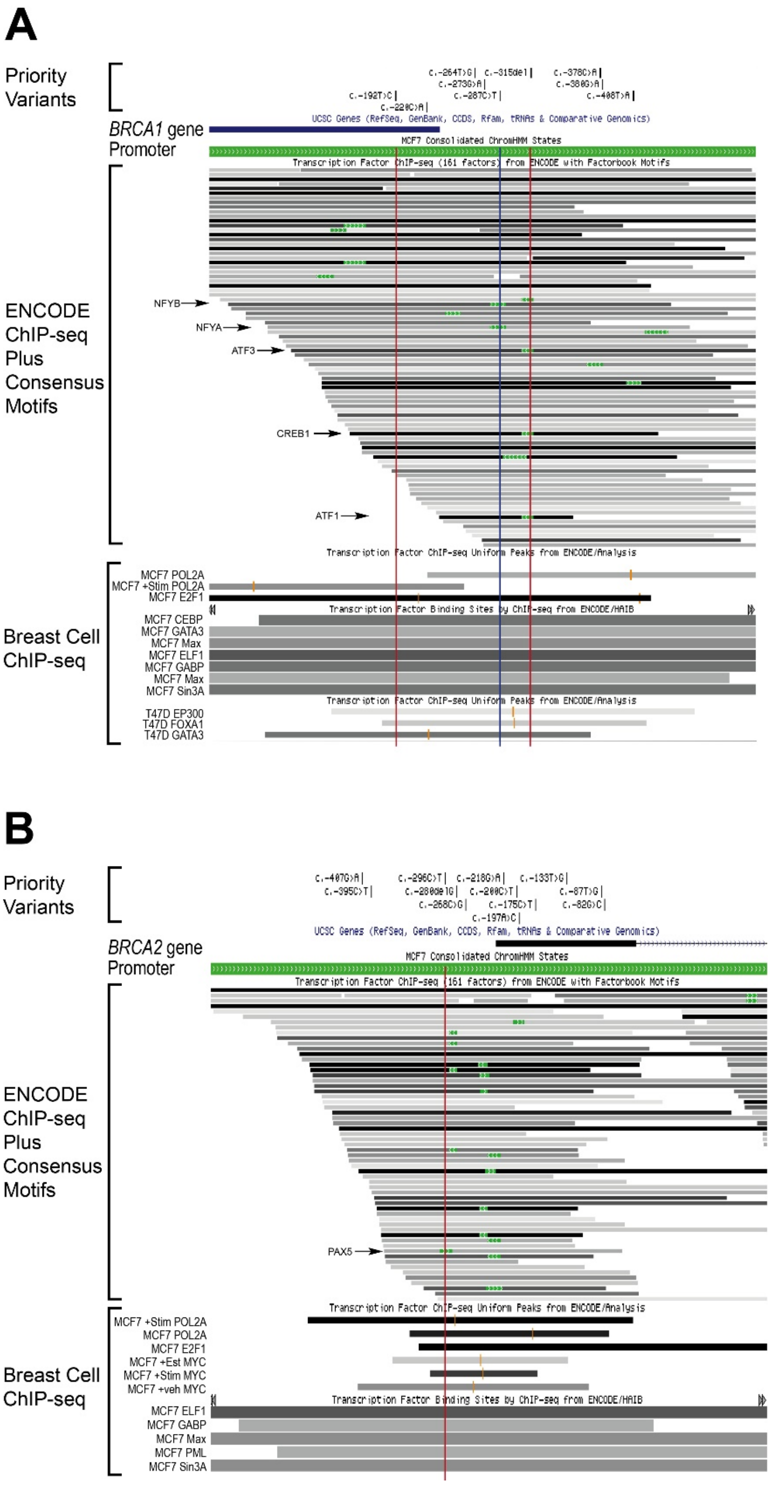

Supplementary Figure S1. Variants in *BRCA1* and *BRCA2* overlap with potential TF binding sites. Snapshots of the UCSC genome browser showing *BRCA1* (A) and *BRCA2* (B) prioritized variants and ENCODE ChIP-seq data from multiple cell lines and available breast cell specific TF ChIPseq data. TF consensus motifs within the ENCODE ChIP-seq dataset tracks are displayed in green. Genomic position of variants that alter luciferase activity are indicated by vertical lines.

Supplementary Table S1.
Oligonucleotides used in this study

Primer Name	5' Forward Primer sequence 3'	5' Reverse Primer sequence 3'
**Promoter Amplification**		
BRCA2-Prom	GCGCTCGAGTGTAAGATCGGCTCGCTTTG	GGCCTCGAGTTGGCGAAAATTTGCCTTCGGGCAAGG
**Site directed Mutagenesis**		
B1-Ets	CTCAGGTAGAATTCTTCCTCTTTTGTCTCTTTCCTTTTACGTCATC	GATGACGTAAAAGGAAAGAGACAAAAGAGGAAGAATTCTACCTGAG
B1:c.-408A	GTACAGCAATTACTGTGAAGCAATAAGCCGCAACTGG	CCAGTTGCGGCTTATTGCTTCACAGTAATTGCTGTAC
B1:c.-380A	CAACTGGAAGAGTAGAAGCTAGAGGGCAGGCAC	GTGCCTGCCCTCTAGCTTCTACTCTTCCAGTTG
B1:c.-378A	CTGGAAGAGTAGAGGATAGAGGGCAGGCACT	AGTGCCTGCCCTCTATCCTCTACTCTTCCAG
B1:c.-356G	GGGCAGGCACTTTATGGGAAACTCAGGTAGAATTC	GAATTCTACCTGAGTTTCCCATAAAGTGCCTGCCC
B1:c.-315del	CGTCTCTTTCCTTTACGTCATCCGGGGG	CCCCCGGATGACGTAAAGGAAAGAGACG
B1:c.-287T	CAGACTGGGTGGCTAATCCAGAGCCCC	GGGGCTCTGGATTAGCCACCCAGTCTG
B1:c.-273A	CCAATCCAGAGCCCCAAGAGACGCTTGGCTC	GAGCCAAGCGTCTCTTGGGGCTCTGGATTGG
B1:c.-264G	GCCCCGAGAGACGCGTGGCTCTTTCTGTC	GACAGAAAGAGCCACGCGTCTCTCGGGGC
B1:c.-220A	CTCTGATTGTACCTTGATTTAGTATTCTGAGAGGCTGCTGC	GCAGCAGCCTCTCAGAATACTAAATCAAGGTACAATCAGAG
B1:c.-192C	CTGCTGCTTAGCGGCAGCCCCTTGGTTTC	GAAACCAAGGGGCTGCCGCTAAGCAGCAG
B1:c.-174T	CCCCTTGGTTTCCGTTGCAACGGAAAAGCGC	GCGCTTTTCCGTTGCAACGGAAACCAAGGGG
B1:c.-171C	CTTGGTTTCCGTGGCCACGGAAAAGCGCGGG	CCCGCGCTTTTCCGTGGCCACGGAAACCAAG
B2-Ets	CCAGGCCTGACTTCCAAGTGGTGCGTGTGCTG	CAGCACACGCACCACTTGGAAGTCAGGCCTGG
B2:c.-407A	CTCTTAAGGGTCAGCGAAAAGAGAACACACACTCC	GGAGTGTGTGTTCTCTTTTCGCTGACCCTTAAGAG
B2:c.-395T	CGAGAAGAGAACACATACTCCAGCTCCCGC	GCGGGAGCTGGAGTATGTGTTCTCTTCTCG
B2:c.-296T	CCGCAGCGGCCCACCTAGGCCTGACTT	AAGTCAGGCCTAGGTGGGCCGCTGCGG
B2:c.-280delG	GCCTGACTTCCGGTGGTGCGTGTGC	GCACACGCACCACCGGAAGTCAGGC
B2:c.-268G	GTGGTGCGTGTGGTGCGTGTCGCGTC	GACGCGACACGCACCACACGCACCAC
B2:c.-218A	GGCTTGTGGCGCGAACTTCTGAAACTAGG	CCTAGTTTCAGAAGTTCGCGCCACAAGCC
B2:c.-200T	CTTCTGAAACTAGGCGGTAGAGGCGGAGCCGCTG	CAGCGGCTCCGCCTCTACCGCCTAGTTTCAGAAG
B2:c.-197C	AAACTAGGCGGCAGCGGCGGAGCCGCTGT	ACAGCGGCTCCGCCGCTGCCGCCTAGTTT
B2:c.-175T	CGCTGTGGCACTGTTGCGCCTCTGCTG	CAGCAGAGGCGCAACAGTGCCACAGCG
B2:c.-133G	TTTTGCGGCGGTGGGGCGCCGCCGGGAGAA	TTCTCCCGGCGGCGCCCCACCGCCGCAAAA
B2:c.-87G	GATTTGTGACCGGCGCGGGTTTTGTCAGCTTACTCCG	CGGAGTAAGCTGACAAAACCCGCGCCGGTCACAAATC
B2:c.-82C	CCGGCGCGGTTTTTCTCAGCTTACTCCGG	CCGGAGTAAGCTGAGAAAAACCGCGCCGG
**Electrophoretic Mobility Shift Assays**		
B1-Ets-WT	AGAATTCTTCCTCTTCCGTCTCTTTCCTTTTA	TAAAAGGAAAGAGACGGAAGAGGAAGAATTCT
B1-Ets-var	AGAATTCTTCCTCTTTTGTCTCTTTCCTTTTA	TAAAAGGAAAGAGACAAAAGAGGAAGAATTCT
B1:c.-315-WT	CGTCTCTTTCCTTTTACGTCATCCGGGGG	CCCCCGGATGACGTAAAAGGAAAGAGACG
B1:c.-315-del	CGTCTCTTTCCTTTACGTCATCCGGGGG	CCCCCGGATGACGTAAAGGAAAGAGACG
B1:c.-287C-WT	GGCAGACTGGGTGGCCAATCCAGAGCCCCGAG	CTCGGGGCTCTGGATTGGCCACCCAGTCTGCC
B1:c.-287T-var	GGCAGACTGGGTGGCTAATCCAGAGCCCCGAG	CTCGGGGCTCTGGATTAGCCACCCAGTCTGCC
B1:c.-192T-WT	GCTGCTGCTTAGCGGTAGCCCCTTGGTTTCC	GGAAACCAAGGGGCTACCGCTAAGCAGCAGC
B1:c.-192C-var	GCTGCTGCTTAGCGGCAGCCCCTTGGTTTCC	GGAAACCAAGGGGCTGCCGCTAAGCAGCAGC
B1:c.-171A-WT	CTTGGTTTCCGTGGCAACGGAAAAGCGCGGG	CCCGCGCTTTTCCGTTGCCACGGAAACCAAG
B1:c.-171C-var	CTTGGTTTCCGTGGCCACGGAAAAGCGCGGG	CCCGCGCTTTTCCGTGGCCACGGAAACCAAG
B1-NS	CACTGGGGCTGAGGGGTGGAACTACGAGTG	CACTCGTAGTTCCACCCCTCAGCCCCAGTG
B2-Ets-WT	CCAGGCCTGACTTCCGGGTGGTGCGTGTGCTG	CAGCACACGCACCACCCGGAAGTCAGGCCTGG
B2-Ets-var	CCAGGCCTGACTTCCAAGTGGTGCGTGTGCTG	CAGCACACGCACCACTTGGAAGTCAGGCCTGG
B2:c.-296C-WT	CCGGAAGTCAGGCCTGGGTGGGCCGCTGCGG	CCGGAAGTCAGGCCTGGGTGGGCCGCTGCGG
B2:c.-296T-var	CCGCAGCGGCCCACCTAGGCCTGACTTCCGG	CCGCAGCGGCCCACCTAGGCCTGACTTCCGG
B2:c.-197A-WT	AAACTAGGCGGCAGAGGCGGAGCCGCTGT	ACAGCGGCTCCGCCTCTGCCGCCTAGTTT
B2:c.-197C-var	AAACTAGGCGGCAGCGGCGGAGCCGCTGT	ACAGCGGCTCCGCCGCTGCCGCCTAGTTT
B2-NS	CTCTACTTCCCTCTTGCGCTTTCTCAATGG	CCATTGAGAAAGCGCAAGAGGGAAGTAGAG
hDAO-PAX5	TCTGTGGGCAGTGCAGTGCCGAGCCCCAC	GTGGGGCTCGGCACTGCACTGCCCACAGA
hCD19-PAX5	AGAATGGGGCCTGAGGCGTGACCACCGCC	GGCGGTGGTCACGCCTCAGGCCCCATTCT
WT- Wildtype		
var- variant		

Supplementary Table S2.
Overview of rare variants in the *BRCA1* 5'upstream region in patients and controls.*

Gene	hg19 Position (chr17)	Variant Name (NM_007294.3)	Site:	Paris	Pisa	Copenhagen	Ghent	Barcelona	Milan		Santiago de Compostela		Prague		*Total screened*		rsID	Global MAF in dbSNP***	TF Motif (ENCODE)	Bioinformatic Priority	Reasons for priority****	Selected for promoter assays
			Samples screened:	Cases	Cases	Cases	Cases	Cases	Cases	Controls	Cases	Controls	Cases	Controls	*Cases*	*Controls*						
			n:	686	80	1157	357	192	772	661	270	130	2961	312	*6475*	*1103*						
BRCA1	g.41278312G>A	c.-1044C>T							1	1			1	0	*2*	*1*	rs554849044	0.00020		Med/Low	Promoter, no open chromatin, BC-TF Low, TF, conserved, conserved element	Minimal promoter - not analyzable
BRCA1	g.41278216C>T	c.-948G>A											2	0	*2*	*0*	Novel			Med/Low	Promoter, no open chromatin, BC-TF Low, TF, conserved, conserved element	Minimal promoter - not analyzable
BRCA1	g.41278214C>T	c.-946G>A			1										*1*	*0*	Novel			Med/Low	Promoter, no open chromatin, BC-TF Low, TF, conserved, conserved element	Minimal promoter - not analyzable
BRCA1	g.41278207G>A	c.-939C>T							1	0					*1*	*0*	rs941238609	0.00007		Med/Low	Promoter, no open chromatin, BC-TF Low, TF, conserved, conserved element	Minimal promoter - not analyzable
BRCA1	g.41278200A>G	c.-932T>C							1	0					*1*	*0*	Novel			Med/Low	Promoter, no open chromatin, BC-TF Low, TF, conserved, conserved element	Minimal promoter - not analyzable
BRCA1	g.41277998G>A	c.-730C>T					2		21	25			54	8	*77*	*33*	rs73625095	0.00519		Med/Low	Promoter, no open chromatin, BC-TF Med, edge of TF, no conservation	Minimal promoter - not analyzable
BRCA1	g.41277944A>T	c.-676T>A					4						41	10	*45*	*10*	rs8176072	0.00120		Med/Low	Promoter, no open chromatin, BC-TF Med, edge of TF, no conservation	Minimal promoter - not analyzable
BRCA1	g.41277861A>G	c.-593T>C					1								*1*	*0*	Novel			Med/Low	Promoter, no open chromatin, BC-TF Med, edge of TF, some conservation	Minimal promoter - not analyzable
BRCA1	g.41277857T>C	c.-589A>G					1						3	0	*4*	*0*	rs563017140	0.00200		Med/Low	Promoter, no open chromatin, BC-TF Med, edge of TF, some conservation	Minimal promoter - not analyzable
BRCA1	g.41277853G>T	c.-585C>A		2			2		5	8	3	0	7	0	*19*	*8*	rs73625094	0.00060		Med/Low	Promoter, no open chromatin, BC-TF Med, edge of TF conserved, conserved element	Minimal promoter - not analyzable
BRCA1	g.41277845T>C	c.-577A>G							1	0					*1*	*0*	Novel			Med/Low	Promoter, no open chromatin, BC-TF Med, edge of TF conserved, conserved element	Minimal promoter - not analyzable
BRCA1	g.41277784T>G	c.-516A>C							1	0					*1*	*0*	Novel			Med/Low	Promoter, no open chromatin, BC-TF High, edge of TF, some conservation	No- Med/Low
BRCA1	g.41277765C>T	c.-497G>A					3								*3*	*0*	rs8176074	0.00220		Med/Low	Promoter, no open chromatin, BC-TF High, edge of TF, some conservation	No- Med/Low
BRCA1	**g.41277676A>T**	**c.-408T>A**			1										*1*	*0*	Novel		CEBP	High/Med	Promoter, some open chromatin, BC-TF High, TF, CEBPB consensus, conserved	Yes
BRCA1	**g.41277648C>T**	**c.-380G>A**							1	0	1	0	1	0	*3*	*0*	Novel		RXRA	High/Med	Promoter, some open chromatin, BC-TF High, TF, RXR consensus, no conservation	Yes
BRCA1	**g.41277646G>T**	**c.-378C>A**					1								*1*	*0*	rs186775935	0.00040	RXRA	High/Med	Promoter, some open chromatin, BC-TF High, TF, RXR consensus, no conservation	Yes
BRCA1	g.41277639C>A	c.-371G>T											1	0	*1*	*0*	novel		RXRA	High/Med	Promoter, some open chromatin, BC-TF High, TF, RXR consensus, no conservation	no - identified after prioritization
BRCA1	g.41277634G>A	c.-366C>T											2	0	*2*	*0*	novel		RXRA	High/Med	Promoter, some open chromatin, BC-TF High, TF, RXR consensus, no conservation	no - identified after prioritization
BRCA1	**g.41277583del**	**c.-315del**						1							*1*	*0*	rs901029407	0.00003	ATF1,2,3***	Med	Promoter, some open chromatin, BC-TF High, TF, some conservation	Yes
BRCA1	**g.41277555G>A**	**c.-287C>T**			1										*1*	*0*	Novel		NFYA, NFYB	High/Med	Promoter, open chromatin, BC-TF High, TF, NFYA, NFYB consensus, no conservation	Yes
BRCA1	**g.41277541C>T**	**c.-273G>A**					4								*4*	*0*	rs112960339	0.00499		Med	Promoter, BC-DHS Peak, open chromatin, BC-TF High, TF, no conservation	Yes
BRCA1	**g.41277532A>C**	**c.-264T>G**											1	0	*1*	*0*	rs904148166	0.00003		Med	Promoter, BC-DHS Peak, open chromatin, BC-TF High, TF, no conservation	Yes
BRCA1	**g.41277488G>T**	**c.-220C>A**						1							*1*	*0*	Novel			Med	Promoter, BC-DHS Peak, open chromatin, BC-TF High, TF, no conservation	Yes
BRCA1	**g.41277460A>G**	**c.-192T>C**					4								*4*	*0*	rs113323025	0.00519	MAZ	Med	Promoter, BC-DHS Peak, open chromatin, BC-TF High, TF, no conservation	Yes
BRCA1	g.41277389C>G	c.-121G>C				1							1	0	*2*	*0*	Novel			Med	Promoter, BC-DHS Peak, some open chromatin, BC-TF High, TF, no conservation	No -only minor open chromatin
BRCA1	g.41277373A>C	c.-105T>G		2							1	0			*3*	*0*	Novel			Med	Promoter, BC-DHS Peak, some open chromatin, BC-TF High, TF, no conservation	No -only minor open chromatin
BRCA1	g.41277354G>A	c.-86C>T		2		1			5	5	4	0	6	2	*18*	*7*	rs143160357	0.00048		Med	Promoter, BC-DHS Peak, some open chromatin, BC-TF High, TF, no conservation	No -Milan case:control
BRCA1	g.41277339C>T	c.-71G>A		7											*7*	*0*	Novel			Med/Low	Promoter, BC-DHS Peak, no open chromatin, BC-TF High, TF, no conservation	No- Med/Low
BRCA1	g.41277334G>C	c.-66C>G				1									*1*	*0*	LOVD			Med/Low	Promoter, BC-DHS Peak, no open chromatin, BC-TF High, TF, no conservation	No- Med/Low
BRCA1	g.41277329G>A	c.-61C>T				1									*1*	*0*	rs549332987	0.00032		Med/Low	Promoter, BC-DHS Peak, no open chromatin, BC-TF High, TF, no conservation	No- Med/Low
BRCA1	g.41277322C>T	c.-54G>A					1								*1*	*0*	LOVD			Med/Low	Promoter, BC-DHS Peak, no open chromatin, BC-TF High, TF, no conservation	No- Med/Low
BRCA1	g.41277308_41277309dup	c.-41_-40dup		2											*2*	*0*	Novel			Med/Low	Promoter, BC-DHS Peak, no open chromatin, BC-TF High, TF, no conservation	No- Med/Low
BRCA1	g.41277292A>G	c.-24T>C		1											*1*	*0*	Novel			Med/Low	Promoter, BC-DHS Peak, no open chromatin, BC-TF High, TF, no conservation	No- Med/Low
BRCA1	g.41277277G>A	c.-20+11C>T				1									*1*	*0*	rs273898672	0.00040		Med/Low	Promoter, BC-DHS Peak, no open chromatin, BC-TF High, TF, no conservation	Minimal promoter - not analyzable
BRCA1	g.41277260C>T	c.-20+28G>A							1	0					*1*	*0*	rs904265019	0.00003		Med/Low	Promoter, BC-DHS Peak, no open chromatin, BC-TF High, TF, no conservation	Minimal promoter - not analyzable
BRCA1	g.41277240C>T	c.-20+48G>A							1	0					*1*	*0*	Novel			Med/Low	Promoter, BC-DHS Peak, no open chromatin, BC-TF High, TF, no conservation	Minimal promoter - not analyzable
BRCA1	g.41277163T>G	c.-20+125A>C											2	0	*2*	*0*	Novel			Med/Low	Promoter, BC-DHS Peak, no open chromatin, BC-TF Med, edge of TF, no conservation	Minimal promoter - not analyzable
BRCA1	g.41277158_41277163delinsG	c.-20+125_-20+130delinsC									1**		2	0	*3*	*0*	Novel			Med/Low	Promoter, BC-DHS Peak, no open chromatin, BC-TF Med, edge of TF, no conservation	Minimal promoter - not analyzable
BRCA1	g.41277157_41277162del	c.-20+126_-20+131del											1	0	*1*	*0*	Novel			Med/Low	Promoter, BC-DHS Peak, no open chromatin, BC-TF Med, edge of TF, no conservation	Minimal promoter - not analyzable
BRCA1	g.41277130C>G	c.-20+158G>C									1**				*1*	*0*	rs558495450	0.00040		Med/Low	Promoter, BC-DHS Peak, no open chromatin, BC-TF Med, edge of TF, no conservation	Minimal promoter - not analyzable
BRCA1	g.41277105del	c.-20+183del											2	0	*2*	*0*	rs1021308877	0.00007		Med/Low	Promoter, BC-DHS Peak, no open chromatin, BC-TF Med, edge of TF, no conservation	Minimal promoter - not analyzable
BRCA1	g.41277075G>T	c.-20+213C>A							1	0					*1*	*0*	Novel			Med/Low	Promoter, no open chromatin, BC-TF Med, edge of TF, no conservation	Minimal promoter - not analyzable
BRCA1	g.41276986C>T	c.-20+302G>A							1	0					*1*	*0*	Novel			Med/Low	Promoter, no open chromatin, BC-TF Med, edge of TF, no conservation	Minimal promoter - not analyzable
BRCA1	g.41276792T>C	c.-20+496A>G											1	0	*1*	*0*	Novel			Med/Low	Promoter, BC-DHS Peak, no open chromatin, BC-TF Low, TF, no conservation	Minimal promoter - not analyzable
BRCA1	g.41276773T>A	c.-20+515A>T											1	0	*1*	*0*	Novel			Med/Low	Promoter, BC-DHS Peak, no open chromatin, BC-TF Low, TF, no conservation	Minimal promoter - not analyzable
BRCA1	g.41276765_41276774del	c.-20+514_-20+523del							2	5			9	0	*11*	*5*	rs879725813	0.00100		Med/Low	Promoter, BC-DHS Peak, no open chromatin, BC-TF Low, TF, no conservation	Minimal promoter - not analyzable
BRCA1	g.41276765_41276769dup	c.-20+519_-20+523dup							3	1	1**		1	1	*5*	*2*	rs573563977	NA		Med/Low	Promoter, BC-DHS Peak, no open chromatin, BC-TF Low, TF, no conservation	Minimal promoter - not analyzable
BRCA1	g.41276765_41276769del	c.-20+519_-20+523del							1	0					*1*	*0*	rs572878542	0.00559		Med/Low	Promoter, BC-DHS Peak, no open chromatin, BC-TF Low, TF, no conservation	Minimal promoter - not analyzable
BRCA1	g.41276763_41276767del	c.-20+521_-20+525del							4	7	10**		22	5	*36*	*12*	rs770929332	0.00240		Med/Low	Promoter, BC-DHS Peak, no open chromatin, BC-TF Low, TF, no conservation	Minimal promoter - not analyzable
BRCA1	g.41276757C>T	c.-20+531G>A		2					0	1	6**		1	0	*9*	*1*	rs146993095	0.00060		Med/Low	Promoter, BC-DHS Peak, no open chromatin, BC-TF Low, TF, no conservation	Minimal promoter - not analyzable
BRCA1	g.41276642G>A	c.-19-510C>T		2											*2*	*0*	rs935325652	0.00003		Med/Low	Promoter, BC-DHS Peak, some open chromatin, BC-TF Med, TF, no conservation	Minimal promoter - not analyzable
BRCA1	g.41276639G>A	c.-19-507C>T		1											*1*	*0*	rs923953208	0.00003		Med/Low	Promoter, BC-DHS Peak, some open chromatin, BC-TF Med, TF, no conservation	Minimal promoter - not analyzable
BRCA1	g.41276609T>G	c.-19-477A>C							2	2			1	0	*3*	*2*	rs375003483	0.00020		Med/Low	Promoter, BC-DHS Peak, some open chromatin, BC-TF Med, TF, no conservation	Minimal promoter - not analyzable
BRCA1	g.41276581T>G	c.-19-449A>C		1											*1*	*0*	Novel			Low	BC-DHS Peak, Some open chromatin, BC-TF Med, TF, no conservation	Minimal promoter - not analyzable
BRCA1	g.41276566_41276582dup	c.-19-450_-19-434dup		1											*1*	*0*	Novel			Low	BC-DHS Peak, Some open chromatin, BC-TF Med, TF, no conservation	Minimal promoter - not analyzable
BRCA1	g.41276565T>C	c.-19-433A>G							0	1			1	0	*1*	*1*	rs191197821	0.00020		Low	BC-DHS Peak, Some open chromatin, BC-TF Med, TF, no conservation	Minimal promoter - not analyzable
BRCA1	g.41276505A>G	c.-19-373T>C											1	0	*1*	*0*	Novel			Low	BC-DHS Peak, No open chromatin, BC-TF Med, TF, no conservation	Minimal promoter - not analyzable
BRCA1	g.41276399_41276401del	c.-19-269_-19-267del											1	0	*1*	*0*	Novel			Low	BC-DHS Peak, No open chromatin, BC-TF Low, edge of TF, no conservation	Minimal promoter - not analyzable
BRCA1	g.41276374T>A	c.-19-242A>T											2	0	*2*	*0*	rs774731955	NA		Low	BC-DHS Peak, No open chromatin, BC-TF Low, edge of TF, no conservation	Minimal promoter - not analyzable
BRCA1	g.41276360C>T	c.-19-228G>A							1	0					*1*	*0*	Novel			Low	BC-DHS Peak, No open chromatin, BC-TF Low, edge of TF, no conservation	Minimal promoter - not analyzable
BRCA1	g.41276354C>T	c.-19-222G>A							1	0					*1*	*0*	rs564004787	0.00020		Low	BC-DHS Peak, No open chromatin, BC-TF Low, edge of TF, no conservation	Minimal promoter - not analyzable
BRCA1	g.41276304C>T	c.-19-172G>A		1											*1*	*0*	Novel			Low	Some open chromatin, BC-TF Low, edge of TF, no conservation	Minimal promoter - not analyzable
BRCA1	g.41276248A>G	c.-19-116T>C		1											*1*	*0*	Novel			Low	Some open chromatin, BC-TF Low, edge of TF, no conservation	Minimal promoter - not analyzable
BRCA1	g.41276241T>G	c.-19-109A>C		1											*1*	*0*	Novel			Low	Some open chromatin, BC-TF Low, edge of TF, no conservation	Minimal promoter - not analyzable
BRCA1	g.41276224T>G	c.-19-92A>C		1											*1*	*0*	Novel			Low	Some open chromatin, BC-TF Low	Minimal promoter - not analyzable
BRCA1	g.41276192C>T	c.-19-60G>A		1											*1*	*0*	Novel			Low	Some open chromatin, BC-TF Low	Minimal promoter - not analyzable
BRCA1	g.41276187del	c.-19-55del		1											*1*	*0*	Novel			Low	Some open chromatin, BC-TF Low	Minimal promoter - not analyzable
BRCA1	g.41276187_41276189del	c.-19-57_-19-55del		1											*1*	*0*	Novel			Low	Some open chromatin, BC-TF Low	Minimal promoter - not analyzable
BRCA1	g.41276182_41276183insTT	c.-19-51_-19-50insAA		1											*1*	*0*	Novel			Low	Some open chromatin, BC-TF Low	Minimal promoter - not analyzable
BRCA1	g.41276171A>T	c.-19-39T>A		1											*1*	*0*	Novel			Low	Some open chromatin, BC-TF Low	Minimal promoter - not analyzable
BRCA1	g.41276161A>G	c.-19-29T>C		2											*2*	*0*	Novel			Low	Some open chromatin, BC-TF Low	Minimal promoter - not analyzable
BRCA1	g.41276156T>C	c.-19-24A>G		3											*3*	*0*	rs370965853	0.00006		Low	Some open chromatin, BC-TF Low	Minimal promoter - not analyzable
BRCA1	g.41276154T>C	c.-19-22A>G		5											*5*	*0*	rs774892235	0.00003		Low	Some open chromatin, BC-TF Low	Minimal promoter - not analyzable
BRCA1	g.41276153A>G	c.-19-21T>C				1									*1*	*0*	Novel			Low	Some open chromatin, BC-TF Low	Minimal promoter - not analyzable
BRCA1	g.41276153_41276154dup	c.-19-22_-19-21dup		8											*8*	*0*	rs273898667	0.00040		Low	Some open chromatin, BC-TF Low	Minimal promoter - not analyzable
BRCA1	g.41276153_41276154del	c.-19-22_-19-21del		1											*1*	*0*	rs564961882	0.00030		Low	Some open chromatin, BC-TF Low	Minimal promoter - not analyzable
BRCA1	g.41276142A>G	c.-19-10T>C		10		1									*11*	*0*	rs201866997	0.00006		Low	Some open chromatin, BC-TF Low	Minimal promoter - not analyzable
BRCA1	g.41276135T>C	c.-19-3A>G		1											*1*	*0*	rs273898669	0.00013		Low	Some open chromatin, BC-TF Low	Minimal promoter - not analyzable
BRCA1	g.41276129T>C	c.-16A>G					1								*1*	*0*	rs777262055	0.00002		Low	Some open chromatin, BC-TF Low	Minimal promoter - not analyzable
BRCA1	g.41276127A>G	c.-14T>C		1											*1*	*0*	rs273897661	NA		Low	Some open chromatin, BC-TF Low, conserved, conserved element	Minimal promoter - not analyzable
BRCA1	g.41276120C>T	c.-7G>A		5											*5*	*0*	Novel			Low	Some open chromatin, BC-TF Low, conserved, conserved element	Minimal promoter - not analyzable

* "Rare" defined as global minor allele frequency <0.01 in dbSNP.
** >1600 cases
*** NA no data available
***- while the variant overlaps with this motif, the deletion does not alter the motif sequence
**** Reason for prioritization:
TF- Transcription Factor Chip-seq peak from all cells tested
BC-TF- Breast Cell specific Transcription Factor Chip-seq peak
BC-DHS Peak- Breast Cell specific DNaseI Hypersensitive Site peak
Variants highlighted in grey were identified in individuals from more than 1 site
Variants in bold were selected for functional assays

Supplementary Table S3.
Overview of rare variants in the *BRCA2* 5'upstream region in patients and controls.*

Gene	hg19 Position (Chr13)	Variant Name (NM_000059.3)	Site:	Paris	Pisa	Copenhagen	Ghent	Barcelona	Maastricht	Santiago de Compostela		Prague		*Total screened*		rsID	Global MAF in dbSNP****	TF Motif (ENCODE)	Bioinformatic Priority	Reasons for priority*****	Selected for promoter assays
			Samples screened:	Cases	Cases	Cases	Cases	Cases	Cases	Cases	Controls	Cases	Controls	*Cases*	*Controls*						
			n:	686	80	1157	357	192	900	270	130	2961	312	*6603*	*442*						
BRCA2	g.32888651C>T	c.-1193C>T			1									*1*	*0*	Novel			Low	Enhancer, some open chromatin, BC-TF Low, no conservation	Minimal promoter - not analyzable
BRCA2	g.32888722T>C	c.-1122T>C					1							*1*	*0*	Novel			Low	Enhancer, some open chromatin, BC-TF Low, no conservation	Minimal promoter - not analyzable
BRCA2	g.32888817C>A	c.-1027C>A					1	1						*2*	*0*	rs11571570	0.0040		Med/Low	Promoter, some open chromatin, BC-TF Low, TF, no conservation	Minimal promoter - not analyzable
BRCA2	g.32888827T>C	c.-1017T>C					1							*1*	*0*	rs552772095	NA		Med/Low	Promoter, some open chromatin, BC-TF Med, TF, no conservation	Minimal promoter - not analyzable
BRCA2	g.32888848dup	c.-996dup					1							*1*	*0*	rs768013835	NA		Med/Low	Promoter, some open chromatin, BC-TF Med, TF, no conservation	Minimal promoter - not analyzable
BRCA2	g.32888920C>A	c.-924C>A			1					2	2			*3*	*2*	rs574423740	0.0014		Med/Low	Promoter, some open chromatin, BC-TF Med, TF, no conservation	Minimal promoter - not analyzable
BRCA2	g.32888975G>A	c.-869G>A					1			0	1			*1*	*1*	rs149257887	0.0080		Med/Low	Promoter, some open chromatin, BC-TF Med, TF, no conservation	Minimal promoter - not analyzable
BRCA2	g.32888990del	c.-854del					1	1						*2*	*0*	rs148073463	NA		Med/Low	Promoter, some open chromatin, BC-TF Med, TF, no conservation	Minimal promoter - not analyzable
BRCA2	g.32889144T>G	c.-700T>G			1					3	2	5	1	*9*	*3*	rs541454524	0.0014		Med/Low	Promoter, BC-DHS Peak, some open chromatin, BC-TF Med, edge of TF, no conservation	Minimal promoter - not analyzable
BRCA2	g.32889172C>G	c.-672C>G					1							*1*	*0*	rs531974275	0.0004		Med/Low	Promoter, BC-DHS Peak, some open chromatin, BC-TF Med, edge of TF, no conservation	Minimal promoter - not analyzable
BRCA2	g.32889214G>T	c.-630G>T								1***	0			*1*	*0*	Novel			Med/Low	Promoter, BC-DHS Peak, some open chromatin, BC-TF Med, edge of TF, no conservation	Minimal promoter - not analyzable
BRCA2	g.32889252G>C	c.-592G>C						1						*1*	*0*	Novel			Med/Low	Promoter, BC-DHS Peak, some open chromatin, BC-TF Med, edge of TF, no conservation	No- Med/Low
BRCA2	g.32889323delG	c.-521delG			2									*2*	*0*	Novel			Med/Low	Promoter, BC-DHS Peak, some open chromatin, BC-TF Med, edge of TF, no conservation	No- Med/Low
BRCA2	g.32889349G>A	c.-495G>A					1							*1*	*0*	rs148060832	0.0002		Med/Low	Promoter, BC-DHS Peak, some open chromatin, BC-TF Med, edge of TF, no conservation	No- Med/Low
BRCA2	g.32889426A>G	c.-418A>G										1	0	*1*	*0*	Novel			Med	Promoter, BC-DHS Peak, some open chromatin, BC-TF High, TF, no conservation	no - identified after prioritization
BRCA2	**g.32889437G>A**	**c.-407G>A**					3			1***	1	38	5	*42*	*6*	rs36221751	0.0018		Med	Promoter, BC-DHS Peak, some open chromatin, BC-TF High, TF, no conservation	Yes
BRCA2	**g.32889449C>T**	**c.-395C>T**										1	0	*1*	*0*	Novel			Med	Promoter, BC-DHS Peak, some open chromatin, BC-TF High, TF, no conservation	Yes
BRCA2	g.32889487T>C	c.-357T>C										1	0	*1*	*0*	Novel			Med	Promoter, BC-DHS Peak, some open chromatin, BC-TF High, TF, no conservation	no - identified after prioritization
BRCA2	**g.32889548C>T**	**c.-296C>T**					1			1***	0	31	5	*33*	*5*	rs563971900	0.0004	PAX5	High/Med	Promoter, BC-DHS Peak, open chromatin, BC-TF High, TF, PAX5 consensus, no conservation	Yes
BRCA2	**g.32889564delG**	**c.-280delG**				1								*1*	*0*	Novel		ELF1, GABPA, ELK1,4	High	Promoter, BC-DHS Peak, open chromatin, BC-TF High, TF, ELF1, ELK1, ELK4 consensus, conserved, conserved element	yes
BRCA2	g.32889567_32889575dup	c.-277_-269dup								1***	0			*1*	*0*	rs762246664	NA		Med	Promoter, BC-DHS Peak, some open chromatin, BC-TF High, TF, some conservation	no - misannotated in first round
BRCA2	**g.32889576C>G**	**c.-268C>G**					1							*1*	*0*	Novel			High/Med	Promoter, BC-DHS Peak, some open chromatin, BC-TF High, TF, conserved	Yes
BRCA2	g.32889593G>A	c.-251G>A										1	0	*1*	*0*	Novel		MAX	High	Promoter, BC-DHS Peak, some open chromatin, BC-TF High, TF, MAX consensus, conserved	no - identified after prioritization
BRCA2	**g.32889626G>A**	**c.-218G>A**								1***	0			*1*	*0*	Novel			Med	Promoter, BC-DHS Peak, some open chromatin, BC-TF High, TF, some conservation	Yes
BRCA2	g.32889627C>T	c.-217C>T							1					*1*	*0*	Novel			Med	Promoter, BC-DHS Peak, some open chromatin, BC-TF High, TF, no conservation	no - identified after prioritization
BRCA2	**g.32889644C>T**	**c.-200C>T**				1								*1*	*0*	Novel		MAZ	Med	Promoter, BC-DHS Peak, open chromatin, BC-TF High, TF, MAZ consensus, no conservation	Yes
BRCA2	**g.32889647A>C**	**c.-197A>C**					1							*1*	*0*	rs370721506	NA	MAZ	Med	Promoter, BC-DHS Peak, open chromatin, BC-TF High, TF, MAZ consensus, no conservation	Yes
BRCA2	**g.32889669C>T**	**c.-175C>T**					7							*7*	*0*	rs55880202	0.0058		Med	Promoter, BC-DHS Peak, open chromatin, BC-TF High, TF, no conservation	Yes
BRCA2	**g.32889711T>G**	**c.-133T>G**				1								*1*	*0*	Novel			Med	Promoter, BC-DHS Peak, no open chromatin, BC-TF High, TF, no conservation	Yes
BRCA2	**g.32889757T>G**	**c.-87T>G**				1								*1*	*0*	Novel			Med/Low	Promoter, BC-DHS Peak, no open chromatin, BC-TF High, edge of TF, no conservation	Yes
BRCA2	**g.32889762G>C**	**c.-82G>C**				1								*1*	*0*	Novel			Med/Low	Promoter, BC-DHS Peak, no open chromatin, BC-TF High, edge of TF, no conservation	Yes
BRCA2	g.32889785_32889787del	c.-59_-57del		1										*1*	*0*	Novel			Med/Low	Promoter, BC-DHS Peak, no open chromatin, BC-TF High, edge of TF, no conservation	No- Med/Low
BRCA2	g.32889811G>T	c.-40+7G>T							2					*2*	*0*	LOVD			Med/Low	Promoter, BC-DHS Peak, no open chromatin, BC-TF High, TF, conservation, conserved element, in splice site region	No- Med/Low/splice site variant
BRCA2	g.32889879C>T	c.-40+75C>T										1	0	*1*	*0*	Novel			Med/Low	Promoter, BC-DHS Peak, some open chromatin, BC-TF Low, edge of TF, no conservation	No- Med/Low
BRCA2	g.32890383C>T	c.-39-176C>T								1***	0			*1*	*0*	Novel			Med/Low	Promoter, BC-DHS Peak, some open chromatin, BC-TF Low, edge of TF, no conservation	Minimal promoter - not analyzable
BRCA2	g.32890443G>A	c.-39-116G>A		1								2	1	*3*	*1*	rs541319330	0.0006		Med/Low	Promoter, some open chromatin, BC-TF Low, edge of TF, no conservation	Minimal promoter - not analyzable
BRCA2	g.32890444G>A	c.-39-115G>A										1	0	*1*	*0*	rs191766418	0.0008		Med/Low	Promoter, some open chromatin, BC-TF Low, edge of TF, no conservation	Minimal promoter - not analyzable
BRCA2	g.32890445A>G	c.-39-114A>G		1										*1*	*0*	rs973070389	NA		Med/Low	Promoter, some open chromatin, BC-TF Low, edge of TF, no conservation	Minimal promoter - not analyzable
BRCA2	g.32890468A>G	c.-39-91A>G		1										*1*	*0*	Novel			Med/Low	Promoter, some open chromatin, BC-TF Low, edge of TF, no conservation	Minimal promoter - not analyzable
BRCA2	g.32890471A>G	c.-39-88A>G		1										*1*	*0*	Novel			Med/Low	Promoter, some open chromatin, BC-TF Low, edge of TF, no conservation	Minimal promoter - not analyzable
BRCA2	g.32890487C>T	c.-39-72C>T		1										*1*	*0*	Novel			Med/Low	Promoter, some open chromatin, BC-TF Low, edge of TF, no conservation	Minimal promoter - not analyzable
BRCA2	g.32890488T>C	c.-39-71T>C		1										*1*	*0*	Novel			Med/Low	Promoter, some open chromatin, BC-TF Low, edge of TF, no conservation	Minimal promoter - not analyzable
BRCA2	g.32890499T>C	c.-39-60T>C		1										*1*	*0*	rs751297272	NA		Med/Low	Promoter, some open chromatin, BC-TF Low, edge of TF, no conservation	Minimal promoter - not analyzable
BRCA2	g.32890523C>G	c.-39-36C>G		2										*2*	*0*	rs183997506	0.0002		Med/Low	Promoter, some open chromatin, BC-TF Low, edge of TF, no conservation	Minimal promoter - not analyzable
BRCA2	g.32890524C>G	c.-39-35C>G		2										*2*	*0*	Novel			Med/Low	Promoter, some open chromatin, BC-TF Low, edge of TF, no conservation	Minimal promoter - not analyzable
BRCA2	g.32890525C>G	c.-39-34C>G		1					1**					*2*	*0*	Novel			Med/Low	Promoter, some open chromatin, BC-TF Low, edge of TF, no conservation	Minimal promoter - not analyzable
BRCA2	g.32890532A>G	c.-39-27A>G		1										*1*	*0*	Novel			Med/Low	Promoter, some open chromatin, BC-TF Low, edge of TF, no conservation	Minimal promoter - not analyzable
BRCA2	g.32890547_32890549del	c.-39-12_-39-10del		14								1	0	*15*	*0*	rs276174798	NA		Med	Promoter, some open chromatin, BC-TF Low, edge of TF, conserved, conserved element	Minimal promoter - not analyzable
BRCA2	g.32890548C>T	c.-39-11C>T		5										*5*	*0*	Novel			Med/Low	Promoter, some open chromatin, BC-TF Low, edge of TF, some conservation	Minimal promoter - not analyzable
BRCA2	g.32890554del	c.-39-5del		1										*1*	*0*	rs276174800	NA		Med	Promoter, some open chromatin, BC-TF Low, edge of TF, conserved, conserved element, in splice site region	Minimal promoter - not analyzable
BRCA2	g.32890555G>T	c.-39-4G>T		3										*3*	*0*	rs765644109	0.0001		Med	Promoter, some open chromatin, BC-TF Low, edge of TF, conserved, conserved element, in splice site region	Minimal promoter - not analyzable
BRCA2	g.32890557A>G	c.-39-2A>G		1										*1*	*0*	LOVD			Med	Promoter, some open chromatin, BC-TF Low, edge of TF, conserved, conserved element, in splice site region	Minimal promoter - not analyzable
BRCA2	g.32890560C>T	c.-38C>T		1										*1*	*0*	Novel			Med	Promoter, some open chromatin, BC-TF Low, edge of TF, no conservation, in splice site region	Minimal promoter - not analyzable
BRCA2	g.32890568C>T	c.-30C>T		2										*2*	*0*	rs775945279	0.000009		Med/Low	Promoter, some open chromatin, BC-TF Low, edge of TF, no conservation	Minimal promoter - not analyzable
BRCA2	g.32890571A>G	c.-27A>G		1										*1*	*0*	rs764618190	NA		Med/Low	Promoter, some open chromatin, BC-TF Low, edge of TF, no conservation	Minimal promoter - not analyzable
BRCA2	g.32890573C>G	c.-25C>G		2										*2*	*0*	LOVD			Med/Low	Promoter, some open chromatin, BC-TF Low, edge of TF, no conservation	Minimal promoter - not analyzable
BRCA2	g.32890583A>C	c.-15A>C		3			1							*4*	*0*	rs138705202	0.0008		Med/Low	Promoter, some open chromatin, BC-TF Low, edge of TF, no conservation	Minimal promoter - not analyzable
BRCA2	g.32890586T>C	c.-12T>C		1										*1*	*0*	rs276174795	NA		Med/Low	Promoter, some open chromatin, BC-TF Low, edge of TF, no conservation	Minimal promoter - not analyzable
BRCA2	g.32890587C>T	c.-11C>T		4			6							*10*	*0*	rs76874770	0.0017		Med/Low	Promoter, some open chromatin, BC-TF Low, edge of TF, no conservation	Minimal promoter - not analyzable
BRCA2	g.32890589T>C	c.-9T>C		2										*2*	*0*	rs276174802	0.000008		Med/Low	Promoter, some open chromatin, BC-TF Low, edge of TF, no conservation	Minimal promoter - not analyzable

* "Rare" defined as global minor allele frequency <0.01 in dbSNP.
** 3300 cases
***276 cases
**** NA no data available
***** Reason for prioritization:
TF- Transcription Factor Chip-seq peak from all cells tested
BC-TF- Breast Cell specific Transcription Factor Chip-seq peak
BC-DHS Peak- Breast Cell specific DNaseI Hypersensitive Site peak
Variants highlighted in grey were identified in individuals from more than 1 site
Variants in bold were selected for functional assays

Supplementary Table S4.
Information Theory Analysis of Prioritized Variants

Variant Name	TFBS Analysis									
	**TF**	**Ri,init**	**Ri,fin**	**TF**	**Ri,init**	**Ri,fin**	**TF**	**Ri,init**	**Ri,fin**	**Consequences**
BRCA1 :c.-408T>A	CEBPB	14.1	11.9	POU5F1	8.5	2.3	STAT1	-2.8	8.2	CEBPB, POU5F1 sites weakened, STAT1 site created
BRCA1 :c.-380G>A	RXRA	4.8	0.5	IRF3	4.5	-0.4	HNF4G	8	1.1	Weak RXRA, IRF3 sites weakened, HNF4G site weakened.
BRCA1 :c.-378C>A	RXRA	4.8	3.2	HSF1	7.7	-0.4	GR	-6.3	7.7	HSF1 site lost and GR site created
BRCA1 :c.-315del	TCF7L2	10.3	-8.1	POU2F2	-20	7.4	IRF4	9.5	12	TCF7L2 site lost and POU2F2 created
BRCA1 :c.-287C>T	NFYA	8.8	-2.1	NFYB	9.1	-2.4	PBX3	-4.8	5.8	NFYA and NFYB sites lost, weak PBX3 site created
BRCA1 :c.-273G>A	ZBTB33	3.9	-6.5	RUNX3	-12.1	5.2				No major changes
BRCA1 :c.-264T>G	BHLHE32	-0.5	7.8	MYC	-1.2	11.2				BHLHE32 and MYC sites created.
BRCA1 :c.-220C>A	RXRA	4	-2	IRF1	5.2	-1.6				No major changes
BRCA1 :c.-192T>C	ETS1	0.4	7.4	RFX5	4.2	10.3				ETS1 site created, weak RFX5 site strengthened.
BRCA2 :c.-407G>A	MEF2A	3.9	7.9	GATA2	5.5	-5.8				Weak MEF2A site srengthened, GATA2 site lost.
BRCA2 :c.-395C>T	TEAD4	8.1	-7.1							TEAD4 site lost.
BRCA2 :c.-296C>T	PAX5	12.5	8.4							PAX5 site weakened .
BRCA2 :c.-280delG	GABPA	12.4	13.5	MXI1	4.8	-4.2	TCF3	7.7	-4	MXI1 andTCF3 sites lost.
BRCA2 :c.-268C>G	BHLHE40	5.1	0	RUNX3	-11.4	6	GR	7.7	5.5	No major changes
BRCA2 :c.-218G>A	TCF3	4.1	1.9	MYC	3.5	-9.2	STAT1	-2.9	2.4	No major changes
BRCA2 :c.-200C>T	KLF1	10.1	-2.5							KLF1 site abolished.
BRCA2 :c.-197A>C	SP4	13.1	4.7	GR	8.9	-5.2	TCF3	-6.6	7.9	SP4 weakened , GR site weakened, TCF3 site created
BRCA2 :c.-175C>T	TCF3	6.5	-1.6	THAP1	3.9	5.8				No major changes
BRCA2 :c.-133T>G	SP4	-5.1	5.2	PAX5	0.6	5.2				No major changes
BRCA2 :c.-87T>G	MXI1	-5.1	5.6	MAFG	4.2	5.4				No major changes
BRCA2 :c.-82G>C	ESR1	4.7	-8.2	IRF3	2.9	9				No major changes

Supplementary Table S5.
Clinical classification of *BRCA1* and *BRCA2* 5' noncoding variants

Gene	Genomic Location (hg19)	HGVS c. Nomenclature	Combined interpretation of frequency information, multifactorial analysis	Highest MAF (population, database)	Prior probability of pathogenicity	Segregation Bayes Score (Number of families)	Tumour histopathology Likelihood ratio (Number of tumours) - See Supp Table 7	Family History	Co-occurrence	Combined odds for causality	Posterior Probability	Multifactorial Classification - 5 Tier IARC class as per Plon et al (2008)	FLOSSIES European MAF	FLOSSIES African MAF	1000 Genomes Admixed American MAF	1000 Genomes East Asian MAF	1000 Genomes Southern Asian MAF	1000 Genomes African MAF	1000 Genomes European MAF	ExAC minus TCGA African MAF	ExAC minus TCGA Admixed American MAF	ExAC minus TCGA East Asian MAF	ExAC minus TCGA Finnish MAF	ExAC minus TCGA Non-Finnish European MAF	ExAC minus TCGA Southern Asian MAF	Milan Controls (This study) Number of Individuals	Milan Controls (This study) MAF	Prague Controls (This study) Number of Individuals	Prague Controls (This study) MAF	Santiago Controls (This study) Number of Individuals	Santiago Controls (This study) MAF
BRCA1	g.41278312G>A	c.-1044C>T	Uncertain	0.001 (European, 1000 Genomes)	0.02										0	0	0	0	0.001							1	0.00076	0	0		
BRCA1	g.41278216C>T	c.-948G>A	Uncertain		0.02																					0	0	0	0		
BRCA1	g.41278214C>T	c.-946G>A	Uncertain		0.02																					0	0	0	0		
BRCA1	g.41278207G>A	c.-939C>T	Uncertain		0.02																					0	0	0	0		
BRCA1	g.41278200A>G	c.-932T>C	Uncertain		0.02																					0	0	0	0		
BRCA1	g.41277998G>A	c.-730C>T	Not Pathogenic[1]	0.01891 (Milan, this study)	0.02										0.0144	0	0	0	0.0159							25	0.01891	8	0.01282		
BRCA1	g.41277944A>T	c.-676T>A	Not Pathogenic[1]	0.01603 (Prague, this study)	0.02										0.0029	0	0	0.0015	0.002							0	0	10	0.01603		
BRCA1	g.41277861A>G	c.-593T>C	Uncertain		0.02																					0	0	0	0		
BRCA1	g.41277857T>C	c.-589A>G	Not Pathogenic[1]	0.0102 (Southern Asian, 1000 Genomes)	0.02										0	0	0.0102	0	0							0	0	0	0		
BRCA1	g.41277853G>T	c.-585C>A	Not Pathogenic[2]	0.00605 (Milan, this study)	0.02	0.0541 (1)				0.0541	0.0011029	likely not pathogenic			0	0	0	0	0.003							8	0.00605	0	0		
BRCA1	g.41277845T>C	c.-577A>G	Uncertain		0.02																					0	0	0	0		
BRCA1	g.41277784T>G	c.-516A>C	Uncertain		0.02																					0	0	0	0		
BRCA1	g.41277765C>T	c.-497G>A	Not Pathogenic[2]	0.0076 (African, 1000 Genomes)	0.02										0.0014	0	0	0.0076	0							0	0	0	0		
BRCA1	g.41277676A>T	c.-408T>A	Uncertain		0.02																					0	0	0	0		
BRCA1	g.41277648C>T	c.-380G>A	Uncertain		0.02		1.67 (1)			1.67	NA															0	0	0	0		
BRCA1	g.41277646G>T	c.-378C>A	Uncertain	0.0015 (African, 1000 Genomes)	0.02										0	0	0	0.0015	0							0	0	0	0		
BRCA1	g.41277639C>A	c.-371G>T	Likely Not Pathogenic		0.02		0.4 (1)			0.4	0.0080972	likely not pathogenic														0	0	0	0		
BRCA1	g.41277634G>A	c.-366C>T	Uncertain		0.02																					0	0	0	0		
BRCA1	g.41277583del	c.-315del	Uncertain		0.02																					0	0	0	0		
BRCA1	g.41277555G>A	c.-287C>T	Uncertain		0.02		0.64 (1)			0.64	NA															0	0	0	0		
BRCA1	g.41277541C>T	c.-273G>A	Not Pathogenic[1]	0.0159 (African, 1000 Genomes)	0.02										0.0058	0	0	0.0159	0							0	0	0	0		
BRCA1	g.41277532A>C	c.-264T>G	Uncertain		0.02		0.51 (1)			0.51	NA															0	0	0	0		
BRCA1	g.41277488G>T	c.-220C>A	Uncertain		0.02																					0	0	0	0		
BRCA1	g.41277460A>G	c.-192T>C	Not Pathogenic[1]	0.0159 (African, 1000 Genomes)	0.02										0.0058	0	0.001	0.0159	0							0	0	0	0		
BRCA1	g.41277389C>G	c.-121G>C	Uncertain	0.00007 (European, FLOSSIES)	0.02		0.59 (1)			0.59	NA		0.00007	0												0	0	0	0		
BRCA1	g.41277373A>C	c.-105T>G	Likely Not Pathogenic		0.02		0.21 (1)			0.21	0.0042674	likely not pathogenic														0	0	0	0		
BRCA1	g.41277354G>A	c.-86C>T	Not Pathogenic	0.00378 (Milan, this study)	0.02	2.0356193 (2)	0.0000108 (8)			0.000022	0.0000005	not pathogenic	0.00089	0						0	0	0	0	0.00077	0.00041	5	0.00378	2	0.00321		
BRCA1	g.41277339C>T	c.-71G>A	Uncertain		0.02																					0	0	0	0		
BRCA1	g.41277334G>C	c.-66C>G	Uncertain		0.02		3.16 (1)			3.16	0.0605828	uncertain														0	0	0	0		
BRCA1	g.41277329G>A	c.-61C>T	Uncertain	0.0008 (African, 1000 Genomes)	0.02								0	0.00078	0	0	0	0.0008	0	0	0	0	0	0	0.00054	0	0	0	0		
BRCA1	g.41277322C>T	c.-54G>A	Uncertain		0.02																					0	0	0	0		
BRCA1	g.41277308_41277309dup	c.-41_-40dup	Uncertain		0.02																					0	0	0	0		
BRCA1	g.41277292A>G	c.-24T>C	Uncertain		0.02																					0	0	0	0		
BRCA1	g.41277277G>A	c.-20+11C>T	Uncertain	0.002 (European, 1000 Genomes)	0.02										0	0	0	0	0.002							0	0	0	0		
BRCA1	g.41277260C>T	c.-20+28G>A	Uncertain		0.02																					0	0	0	0		
BRCA1	g.41277240C>T	c.-20+48G>A	Uncertain		0.02																					0	0	0	0		
BRCA1	g.41277163T>G	c.-20+125A>C	Uncertain		0.02																					0	0	0	0		
BRCA1	g.41277158_41277163delinsG	c.-20+125_-20+130delins	Uncertain		0.02																					0	0	0	0		
BRCA1	g.41277157_41277162del	c.-20+126_-20+131del	Uncertain		0.02																					0	0	0	0		
BRCA1	g.41277130C>G	c.-20+158G>C	Uncertain		0.02																					0	0	0	0		
BRCA1	g.41277105del	c.-20+183del	Uncertain		0.02																					0	0	0	0		
BRCA1	g.41277075G>T	c.-20+213C>A	Uncertain		0.02																					0	0	0	0		
BRCA1	g.41276986C>T	c.-20+302G>A	Uncertain		0.02																					0	0	0	0		
BRCA1	g.41276792T>C	c.-20+496A>G	Uncertain		0.02																					0	0	0	0		
BRCA1	g.41276773T>A	c.-20+515A>T	Uncertain		0.02		4.13 (1)			4.13	0.0777339	uncertain														0	0	0	0		
BRCA1	g.41276765_41276774del	c.-20+514_-20+523del	Not Pathogenic[2]	0.00378 (Milan, this study)	0.02																					5	0.00378	0	0		
BRCA1	g.41276765_41276769dup	c.-20+519_-20+523dup	Uncertain	0.0016 (Prague, this study)	0.02																					1	0.00076	1	0.0016		
BRCA1	g.41276765_41276769del	c.-20+519_-20+523del	Not Pathogenic[1]	0.0166 (African, 1000 Genomes)	0.02										0.0043	0.001	0	0.0166	0.002							0	0	0	0		
BRCA1	g.41276763_41276767del	c.-20+521_-20+525del	Not Pathogenic[2]	0.00801 (Prague, this study)	0.02		1.54 (1)			1.54	NA															7	0.0053	5	0.00801		
BRCA1	g.41276757C>T	c.-20+531G>A	Uncertain	0.002 (European, 1000 Genomes)	0.02										0.0014	0	0	0	0.002							1	0.00076	0	0		
BRCA1	g.41276642G>A	c.-19-510C>T	Uncertain		0.02																					0	0	0	0		
BRCA1	g.41276639G>A	c.-19-507C>T	Uncertain		0.02																					0	0	0	0		
BRCA1	g.41276609T>G	c.-19-477A>C	Uncertain	0.00151 (Milan, this study)	0.02										0	0	0.001	0	0							2	0.00151	0	0		
BRCA1	g.41276581T>G	c.-19-449A>C	Uncertain		0.02																					0	0	0	0		
BRCA1	g.41276566_41276582dup	c.-19-450_-19-434dup	Uncertain		0.02																					0	0	0	0		
BRCA1	g.41276565T>C	c.-19-433A>G	Uncertain	0.001 (European, 1000 Genomes)	0.02										0	0	0	0	0.001							1	0.00076	0	0		
BRCA1	g.41276505A>G	c.-19-373T>C	Uncertain		0.02		3.16 (1)			3.16	0.0605828	uncertain														0	0	0	0		
BRCA1	g.41276399_41276401del	c.-19-269_-19-267del	Uncertain		0.02																					0	0	0	0		
BRCA1	g.41276374T>A	c.-19-242A>T	Uncertain		0.02																					0	0	0	0		
BRCA1	g.41276360C>T	c.-19-228G>A	Uncertain		0.02																					0	0	0	0		
BRCA1	g.41276354C>T	c.-19-222G>A	Uncertain		0.02																					0	0	0	0		
BRCA1	g.41276304C>T	c.-19-172G>A	Uncertain		0.02																					0	0	0	0		
BRCA1	g.41276248A>G	c.-19-116T>C	Uncertain		0.02																					0	0	0	0		
BRCA1	g.41276241T>G	c.-19-109A>C	Uncertain		0.02																					0	0	0	0		
BRCA1	g.41276224T>G	c.-19-92A>C	Uncertain		0.02																					0	0	0	0		
BRCA1	g.41276192C>T	c.-19-60G>A	Uncertain		0.02																					0	0	0	0		
BRCA1	g.41276187del	c.-19-55del	Uncertain		0.02																					0	0	0	0		
BRCA1	g.41276187_41276189del	c.-19-57_-19-55del	Uncertain		0.02																					0	0	0	0		
BRCA1	g.41276182_41276183insTT	c.-19-51_-19-50insAA	Uncertain		0.02																					0	0	0	0		
BRCA1	g.41276171A>T	c.-19-39T>A	Uncertain		0.02																					0	0	0	0		
BRCA1	g.41276161A>G	c.-19-29T>C	Uncertain		0.02																					0	0	0	0		
BRCA1	g.41276156T>C	c.-19-24A>G	Uncertain	0.00065 (African, ExAC)	0.02															0.00065	0	0	0	0.00004	0	0	0	0	0		
BRCA1	g.41276154T>C	c.-19-22A>G	Uncertain		0.02																					0	0	0	0		
BRCA1	g.41276153A>G	c.-19-21T>C	Uncertain		0.02																					0	0	0	0		
BRCA1	g.41276153_41276154dup	c.-19-22_-19-21dup	Not Pathogenic[2]	0.00193 (African, ExAC)	0.02															0.00193	0.00015	0	0.00023	0.00034	0.00017	0	0	0	0		
BRCA1	g.41276153_41276154del	c.-19-22_-19-21del	Uncertain	0.00096 (African, ExAC)	0.02															0.00096	0.00046	0	0	0.0003	0.00008	0	0	0	0		
BRCA1	g.41276142A>G	c.-19-10T>C	Not Pathogenic[3]	0.0008 (African, 1000 Genomes)	0.34	0.0626 (1)[5]		2.1144495	0.2383744	0.0315523	0.0159943	likely not pathogenic	0.0002	0	0	0	0	0.0008	0	0.00026	0.00011	0.00015	0	0	0	0	0	0	0		
BRCA1	g.41276135T>C	c.-19-3A>G	Uncertain	0.00166 (East Asian, ExAC)	0.97										0	0	0.001	0	0	0	0	0.00166	0	0	0.00007	0	0	0	0		
BRCA1	g.41276129T>C	c.-16A>G	Uncertain	0.00023 (African, ExAC)	0.02								0	0.0002						0.00023	0	0	0	0	0	0	0	0	0		
BRCA1	g.41276127A>G	c.-14T>C	Uncertain		0.02			1.033092037	15.42266644	15.9330339	0.2453764	uncertain														0	0	0	0		
BRCA1	g.41276120C>T	c.-7G>A	Uncertain		0.02																					0	0	0	0		
BRCA2	g.32888651C>T	c.-1193C>T	Uncertain		0.02																							0	0	0	0
BRCA2	g.32888722T>C	c.-1122T>C	Uncertain		0.02																							0	0	0	0
BRCA2	g.32888817C>A	c.-1027C>A	Not Pathogenic[1]	0.0151 (African, 1000 Genomes)	0.02										0	0	0	0.0151	0									0	0	0	0
BRCA2	g.32888827T>C	c.-1017T>C	Uncertain		0.02																							0	0	0	0
BRCA2	g.32888848dup	c.-996dup	Uncertain		0.02																							0	0	0	0
BRCA2	g.32888920C>A	c.-924C>A	Uncertain	0.00769 (Santiago de Compostela, this study)	0.02										0.0029	0	0.0031	0.0008	0.001									0	0	2	0.00769
BRCA2	g.32888975G>A	c.-869G>A	Not Pathogenic[1]	0.0295 (African, 1000 Genomes)	0.02										0.0014	0	0	0.0295	0									0	0	1	0.00385
BRCA2	g.32888990del	c.-845del	Uncertain		0.02																							0	0	0	0
BRCA2	g.32889144T>G	c.-700T>G	Uncertain	0.00769 (Santiago de Compostela, this study)	0.02		1.07 (1)			1.07	NA				0.0029	0	0.0031	0.0008	0.001									1	0.0016	2	0.00769
BRCA2	g.32889172C>G	c.-672C>G	Uncertain	0.0015 (African, 1000 Genomes)	0.02										0	0	0	0.0015	0									0	0	0	0
BRCA2	g.32889214G>T	c.-630G>T	Uncertain		0.02																							0	0	0	0
BRCA2	g.32889252G>C	c.-592G>C	Uncertain		0.02																							0	0	0	0
BRCA2	g.32889323delG	c.-521del	Uncertain		0.02																							0	0	0	0
BRCA2	g.32889349G>A	c.-495G>A	Uncertain	0.0008 (African, 1000 Genomes)	0.02										0	0	0	0.0008	0									0	0	0	0
BRCA2	g.32889426A>G	c.-418A>G	Uncertain		0.02																							0	0	0	0
BRCA2	g.32889437G>A	c.-407G>A	Not Pathogenic[2]	0.00801 (Prague, this study)	0.02		0.55162793 (6)			0.55162793	NA				0.0029	0	0.001	0	0.006									5	0.00801	1	0.00385
BRCA2	g.32889449C>T	c.-395C>T	Uncertain		0.02																							0	0	0	0
BRCA2	g.32889487T>C	c.-357T>C	Uncertain		0.02																							0	0	0	0
BRCA2	g.32889548C>T	c.-296C>T	Not Pathogenic[2]	0.00801 (Prague, this study)	0.02	3.0731 (1)	1.9090427 (8)			5.8666792	0.1069261	uncertain			0	0	0	0	0.002									5	0.00801	0	0
BRCA2	g.32889564delG	c.-280del	Uncertain		0.02		0.69 (1)			0.69	NA																	0	0	0	0
BRCA2	g.32889567_32889575dup	c.-277_-269dup	Likely Not Pathogenic		0.02	0.1907 (1)	0.37 (1)			0.070559	0.0014379	likely not pathogenic																0	0	0	0
BRCA2	g.32889576C>G	c.-268C>G	Uncertain		0.02																							0	0	0	0
BRCA2	g.32889593G>A	c.-251G>A	Uncertain		0.02																							0	0	0	0
BRCA2	g.32889626G>A	c.-218G>A	Likely Not Pathogenic		0.02	0.5242 (1)	0.72 (1)			0.377424	0.0076437	likely not pathogenic																0	0	0	0
BRCA2	g.32889627C>T	c.-217C>T	Uncertain	0.00007 (European, FLOSSIES)	0.02								0.00007	0														0	0	0	0
BRCA2	g.32889644C>T	c.-200C>T	Likely Not Pathogenic		0.02		0.37 (1)			0.37	0.0074944	likely not pathogenic																0	0	0	0
BRCA2	g.32889647A>C	c.-197A>C	Not Pathogenic[2]	0.00137 (African, FLOSSIES)	0.02		1.08 (1)			1.08	NA		0	0.00137														0	0	0	0
BRCA2	g.32889669C>T	c.-175C>T	Not Pathogenic[1]	0.01973 (African, FLOSSIES)	0.02								0.00096	0.01973	0.0058	0	0	0.0189	0									0	0	0	0
BRCA2	g.32889711T>G	c.-133T>G	Uncertain		0.02																							0	0	0	0
BRCA2	g.32889757T>G	c.-87T>G	Uncertain		0.02																							0	0	0	0
BRCA2	g.32889762G>C	c.-82G>C	Uncertain		0.02																							0	0	0	0
BRCA2	g.32889785_32889787del	c.-59_-57del	Not Pathogenic[1]	0.0307 (Southern Asian, 1000 Genomes)	0.02										0	0	0.0307	0	0									0	0	0	0
BRCA2	g.32889811G>T	c.-40+7G>T	Uncertain		0.02																							0	0	0	0
BRCA2	g.32889879C>T	c.-40+75C>T	Uncertain		0.02																							0	0	0	0
BRCA2	g.32890383C>T	c.-39-176C>T	Uncertain		0.02		1.08 (1)			1.08	NA																	0	0	0	0
BRCA2	g.32890443G>A	c.-39-116G>A	Uncertain	0.0031 (Southern Asian, 1000 Genomes)	0.02										0	0	0.0031	0	0									1	0.0016	0	0
BRCA2	g.32890444G>A	c.-39-115G>A	Uncertain	0.004 (East Asian, 1000 Genomes)	0.02										0	0.004	0	0	0									0	0	0	0
BRCA2	g.32890445delinsG	c.-39-114A>G	Uncertain		0.02																							0	0	0	0
BRCA2	g.32890468A>G	c.-39-91A>G	Uncertain		0.02																							0	0	0	0
BRCA2	g.32890471A>G	c.-39-88A>G	Uncertain		0.02																							0	0	0	0
BRCA2	g.32890487C>T	c.-39-72C>T	Uncertain		0.02																							0	0	0	0
BRCA2	g.32890488T>C	c.-39-71T>C	Uncertain		0.02																							0	0	0	0
BRCA2	g.32890499T>C	c.-39-60T>C	Uncertain		0.02																							0	0	0	0
BRCA2	g.32890523C>G	c.-39-36C>G	Uncertain	0.0014 (Admixed American, 1000 Genomes)	0.02	0.4456 (1)	1.54 (1)			0.686224	NA				0.0014	0	0	0	0									0	0	0	0
BRCA2	g.32890524C>G	c.-39-35C>G	Uncertain		0.02																							0	0	0	0
BRCA2	g.32890525C>G	c.-39-34C>G	Uncertain		0.02																							0	0	0	0
BRCA2	g.32890532A>G	c.-39-27A>G	Uncertain		0.02																							0	0	0	0
BRCA2	g.32890547_32890549del	c.-39-12_-39-10del	Uncertain		0.34			2.255368	1.4401678	3.2481083	0.625926	uncertain																0	0	0	0
BRCA2	g.32890548C>T	c.-39-11C>T	Uncertain		0.04																							0	0	0	0
BRCA2	g.32890554del	c.-39-5del	Uncertain		0.04			0.917713	1.12928709	1.03636147	NA																	0	0	0	0
BRCA2	g.32890555G>T	c.-39-4G>T	Uncertain	0.00045 (Southern Asian, ExAC)	0.04															0	0.00009	0	0	0	0.00045			0	0	0	0
BRCA2	g.32890557A>G	c.-39-2A>G	Likely Pathogenic[4]		0.97																							0	0	0	0
BRCA2	g.32890560C>T	c.-38C>T	Uncertain		0.04																							0	0	0	0
BRCA2	g.32890568C>T	c.-30C>T	Uncertain	0.00002 (Non-Finnish European, ExAC)	0.02															0	0	0	0	0.00002	0			0	0	0	0
BRCA2	g.32890571A>G	c.-27A>G	Uncertain		0.02																							0	0	0	0
BRCA2	g.32890573C>G	c.-25C>G	Uncertain		0.02																							0	0	0	0
BRCA2	g.32890583A>C	c.-15A>C	Not Pathogenic[2]	0.00293 (African, FLOSSIES)	0.02			0.93514859	2.07408316	1.93957594	NA		0	0.00293	0	0	0	0.003	0	0.00214	0.00018	0	0	0	0			0	0	0	0
BRCA2	g.32890586T>C	c.-12T>C	Uncertain		0.3			0.78980404	1.04983669	0.82916526	NA																	0	0	0	0
BRCA2	g.32890587C>T	c.-11C>T	Not Pathogenic[1]	0.01766 (African, ExAC)	0.02										0	0	0	0.0166	0	0.01766	0.00072	0	0	0	0			0	0	0	0
BRCA2	g.32890589T>C	c.-9T>C	Uncertain	0.00007 (European, FLOSSIES)	0.64	3.65000008 (2)[6]		0.11497767	1.40556914	0.58987311	NA		0.00007	0						0	0	0	0	0.00002	0			0	0	0	0

NA, not applicable. Multifactorial classification was not assigned as the combined odds of causality were insufficient (≥0.5 and ≤2) to derive a posterior probability of pathogenicity (Vallee et al., 2016).

[1]Not pathogenic based on frequency >1% in an outbred sampleset. Segregation bayes scores and tumour histopathology likelihood ratios were not calculated for these variants as the multifactorial likelihood model has been calibrated for rare variants.

[2]Variant allele assigned a low prior probability of pathogenicity of 0.02 assuming conservatively that 2/100 of such variants might be associated with a high risk of cancer AND allele frequency ≥0.001 and <0.01 (>=5 alleles) in outbred sampleset.

[3]Variant allele does not alter splicing in vitro (Fetzer et al 1999) AND co-occurrence in trans with pathogenic variant BRCA1 c.2299delA; p.(Ser767Alafs*25), in a patient with with ovarian cancer at 43 y, reported in Fetzer et al 1999.

[4]Consensus donor/acceptor site variant allele with high likelihood to result in splicing aberration with pathogenic consequences.

[5]Bayes score calculated from family published in Fetzer et al 1999.

[6]Bayes score extracted from previously published dataset (Easton et al,. 2007).

Supplementary Table S6.
Tumour histopathology status for *BRCA1* and *BRCA2* 5' noncoding variants

Gene	Genomic Location (hg19)	HGVS c. Nomenclature	Breast Cancer Diagnosis Age	Breast Cancer Grade	ER Status	PR Status	HER2 Status	Tumour histopathology Likelihood ratio	Total tumour histopathology Likelihood ratio for variant	Total number of tumours for variant
BRCA1	g.41277648C>T	c.-380G>A	28	3	NA	NA	NA	1.67	1.67	1
BRCA1	g.41277639C>A	c.-371G>T	36.9	NA	+	+	+	0.4	0.4	1
BRCA1	g.41277555G>A	c.-287C>T	38	3	+	+	+	0.64	0.64	1
BRCA1	g.41277532A>C	c.-264T>G	60.8	1	-	-	-	0.51	0.51	1
BRCA1	g.41277389C>G	c.-121G>C	31.4	1	-	-	+	0.59	0.59	1
BRCA1	g.41277373A>C	c.-105T>G	35	2	+	+	+	0.21	0.21	1
BRCA1	g.41277354G>A	c.-86C>T	56	3	-	+	-	4.13	0.0000108	8
			41	2	+	+	-	0.21		
			35	2	+	+	-	0.21		
			42	2	+	+	-	0.21		
			33	1	+	+	NA	0.08		
			48	1	+	+	-	0.08		
			42	2	+	-	+	0.21		
			40	2	+	NA	-	0.21		
BRCA1	g.41277334G>C	c.-66C>G	49	3	-	+	-	3.16	3.16	1
BRCA1	g.41276773T>A	c.-20+515A>T	55.2	3	-	-	-	4.13	4.13	1
BRCA1	g.41276763_41276767del	c.-20+521_-20+525delAAAAA	53.8	3	-	-	+	1.54	1.54	1
BRCA1	g.41276505A>G	c.-19-373T>C	33.9	3	-	-	-	3.16	3.16	1
BRCA2	g.32889144T>G	c.-700T>G	41	2	+	+	-	1.07	1.07	1
BRCA2	g.32889437G>A	c.-407G>A	42	2	NA	NA	NA	0.88	0.55162793	6
			34.9	2	+	+	-	1.07		
			34	2	+	-	-	1.07		
			36	3	-	-	+	0.69		
			35.1	3	-	-	-	0.69		
			40.3	NA	+	+	NA	1.15		
BRCA2	g.32889548C>T	c.-296C>T	27	2	+	+	+	1.07	1.9090427	8
			53.8	3	-	-	+	1.54		
			59.7	3	-	-	-	1.54		
			43	2	+	+	-	1.07		
			33.9	3	-	-	-	0.69		
			47.2	2	+	+	-	1.07		
			40	2	+	+	-	1.07		
			61	2	+	NA	-	0.89		
BRCA2	g.32889564del	c.-280del	47	3	-	NA	-	0.69	0.69	1
BRCA2	g.32889567_32889575dup	c.-277_-269dup	45	1	+	+	+	0.37	0.37	1
BRCA2	g.32889626G>A	c.-218G>A	35	NA	-	-	-	0.72	0.72	1
BRCA2	g.32889644C>T	c.-200C>T	43	1	+	NA	-	0.37	0.37	1
BRCA2	g.32889647A>C	c.-197A>C	39	3	NA	NA	NA	1.08	1.08	1
BRCA2	g.32890383C>T	c.-39-176C>T	28	3	NA	NA	NA	1.08	1.08	1
BRCA2	g.32890523C>G	c.-39-36C>G	56	3	-	+	-	1.54	1.54	1
