# Supplementary material for: BRCA1 and BRCA2 5′ noncoding region variants identified in breast cancer patients alter promoter activity and protein binding
Source: Hum Mutat. 2018 Sep 24;39(12):2025–39. doi: 10.1002/humu.23652 (PMC6282814; doi:10.1002/humu.23652)
Supplement: Supplementary file 2 — Supplementary methods [file HUMU-39-2025-s002.pdf]

Burke et al

Supplementary methods

Bioinformatic analysis of variants

Mapping to active regulatory elements

Rare variants mapping to 5' non-coding regions of *BRCA1* and *BRCA2* were assessed for their potential to affect gene regulation using publically available datasets in the University of California, Santa Cruz (UCSC) Genome Browser (hg19) (Kent et al., 2002) and breast cell specific chromatin segregation states. Cell specific signatures have been shown to increase the accuracy of prediction of the effects of enhancer variants (Lee et al., 2015) Therefore analyses and prioritization were restricted to breast cell specific datasets where available. Chromatin segregation datasets for breast cells HMEC and MCF7 (Taberlay, Statham, Kelly, Clark, & Jones, 2014) were used to define the promoter region of *BRCA1* and *BRCA2* (Figure 2). Breast cell specific regulatory marks and TF binding were interrogated using Encyclopedia of DNA Elements (ENCODE; (Dunham, Kundaje, Aldred, & Consortium, 2012) datasets from HMEC, MCF7 and T47D cells. Open chromatin was examined using breast cell specific DNase1 hypersensitivity (DHS) and formaldehyde-assisted isolation of regulatory elements (FAIRE) data sets. Histone H3 epigenetic marks were investigated using Histone ChIP-seq data sets for H3K4me1, H3K4me2, H3K4me3, H3K9ac and H3K27ac from HMEC, MCF7 and T47D cells. Active promoters typically contain increased H3K4me3, H3K27ac and H3K9ac and lack H3K4me1 (Ernst & Kellis, 2010).

Potential active promoter regions were identified as regions containing promoter specific epigenetic marks, breast cell specific DNase1 peaks and multiple breast cell specific TF-ChIP seq peaks. Variants within these active promoter regions were then prioritized for possible functional assay using tracks within the UCSC browser (hg19). Analysis of open chromatin used ENCODE FAIRE and DNaseI peaks and Base Overlap Signal tracks. ChIP-seq data showing TF occupancy and TF consensus motifs in cells other than breast were investigated using TF ChIP-seq V3 from ENCODE. TF binding was investigated using TF ChIP-seq V2 and 3 and breast cell specific TF binding by Transcription Factor Binding Sites by ChIP-seq from ENCODE/HAIB. Conservation was determined using phastCons 46wayPlacental track

and placental mammal Conserved Elements datasets. Scores and features overlapping with variants were analyzed using the Data Integrator within the UCSC Genome Browser.

In silico transcription factor binding and RNA structure analysis

All rare variants were analyzed in silico using an information-theory (IT) based method to assess potential effects of variants on TF binding (Caminsky et al., 2016; Mucaki et al., 2016). A modified version of the Shannon pipeline utilizing TF information models built from ENCODE ChIP-seq data sets (Lu, Mucaki, & Rogan, 2017) determined changes in information content measured in bits at binding sites within chromatin accessible intervals. Differences in the binding affinities of sites overlapping reference (WT) and variant sequences were flagged as significant when the information changes were severe, ie. $\geq$ 4 bits. Weak binding sites with low binding affinity either created by the variant or preceding the sequence change were filtered out by eliminating sites with R_i values at least 1 standard deviation below $R_{sequence}$, the mean information content, of the TFBS information model.

A subset of 5'UTR variants that showed changes in luciferase activity were analyzed for changes in RNABP factor binding using RNAReg2.0 (Chang et al., 2013); http://regrna2.mbc.nctu.edu.tw) and RNA secondary structure by Mfold (http://unafold.rna.albany.edu/?q=mfold) and RNAReg2.0.

Experimental analysis of variants

Cell transfection

Adherent BC derived cell lines MCF7 (ATCC# HTB-22) and MDA-MB-468 (ATCC# HTB-132) were cultured as recommended by ATCC. Cells were transfected with 500ng of the reporter plasmid DNA and 20ng of pRL-TK (Renilla luciferase) DNA using Lipofectamine 3000 (Life Technologies) according to the manufacturer's instructions. Positive controls were B1-Ets, *BRCA1*:c.-330_-329delinsTT, that decreases *BRCA1* promoter activity in MCF7 cells (Atlas, Stramwasser, Whiskin, & Mueller, 2000) and B2-Ets (E2Fmut1: *BRCA2*:c.-282_-281delinsAA), that decreases *BRCA2* promoter activity in MCF7 cells (Davis, Miron, Andersen, Iglehart, & Marks, 1999). pGL3-promoter (Promega) was used as an internal control. Internal control, positive control, WT reporter and reporters containing the variants were prepared simultaneously and transfected on the same plate. Biological replicates were

generated using independent batches of cells cultured and transfected at three separate times with independently prepared batches of plasmids.

Electrophoretic mobility shift assay (EMSA)

Nuclear proteins were extracted from MCF7 and MDA-MB-468 cells using the NE-PER Nuclear and Cytoplasmic Extraction Reagents Kit (Thermofisher, USA) as per the manufacturer's instructions, with the addition of Roche cØmplete, EDTA-free Protease Inhibitor Cocktail (Sigma-Aldrich, Australia). Supernatants containing the nuclear extracts were aliquoted and stored at -80°C. Double stranded oligonucleotides prepared by annealing complementary oligonucleotides containing either the WT or variant allele sequence (Integrated DNA Technologies, Coralville, Iowa; Supp. Table 1) were labeled with biotin using a Pierce Biotin 3' End DNA Labeling Kit (Thermofisher, USA) and purified using a QIAquick Nucleotide Removal Kit (Qiagen). For EMSA binding reactions, nuclear extracts were incubated with biotinylated, double stranded (ds) oligonucleotide probes for 20 min. at 25°C in a total of 20 µl binding buffer (50 mM KCl, 10 mM Tris-HCl pH 7.5, 50 ng/µl dI:dC,0.05% NP-40, 1 mM DTT). Binding reactions were resolved by gel electrophoresis and transferred to charged nylon membranes (Biorad). Biotin-labeled DNA was detected using a Pierce LightShift Chemiluminescent EMSA Kit (Thermofisher, USA) as per the manufacturer's instructions. Competition experiments were carried out by incubating ds competitor probes with nuclear extracts on ice for 10 min before addition of the biotinylated probe and incubation at 25°C for 20 min. For supershift experiments, rabbit monoclonal antibodies to NFYA (EPR9061), Oct-2 (POU2F2; EPR12482) and PAX5 (EPR3730(2)) (Abcam) were incubated with nuclear extracts for 20 min at 25°C in modified binding buffer (50 mM KCl, 10 mM Tris-HCl pH 7.5, 50 ng/µl dI:dC) before addition of the biotinylated probe.

Qualitative and quantitative classification of variants

Population frequency

Population frequencies were obtained from the 1000 Genomes database (Auton et al., 2015), ExAC (excluding TCGA) (Lek et al., 2016), and Fabulous Ladies Over Seventy (FLOSSIES) (http://whi.color.com) datasets. Population frequencies were also calculated from control datasets sequenced as part of this study. Variants were also checked for deposition in other databases including Leiden Open Variation Database (LOVD; http://www.lovd.nl/3.0/home)

at Leiden University Medical Center, ClinVar (https://www.ncbi.nlm.nih.gov/clinvar/intro/) at National Center for Biotechnology Information and BRCAExchange (http://brcaexchange.org/).

Clinical data

Clinical data, where available, included segregation of variants with cancer in families, breast tumour pathology status (estrogen receptor (ER), progesterone receptor (PR), epidermal growth factor receptor 2 (HER2), tumour grade) for variant carriers, and patient phenotype (cancer status, age at onset/interview).

Multifactorial likelihood analysis

Bayes scores for segregation were derived as described previously (Thompson, Easton, & Goldgar, 2003) for seven informative families submitted by participating sites, and three informative families reported in publications (Easton et al., 2007; Fetzer, Tworek, Piver, & DiCioccio, 1999). Likelihood ratios (LRs) for histopathological features were applied according to (Spurdle et al., 2014). Pathology datapoints were excluded if the variant under study co-occurred with an unclassified variant in the same gene. LRs previously computed for variants based on family history or co-occurrence were extracted from a published dataset (Easton et al., 2007). Segregation, histopathology, family history and co-occurrence LRs are mutually independent, and were multiplied to derive a combined odds for causality. The prior probability of pathogenicity was assigned, as described previously (Tavtigian, Byrnes, Goldgar, & Thomas, 2008; Vallee et al., 2016). Variants upstream of the exon 2 acceptor sites (-20) were assigned a prior of 0.02, assuming very conservatively that 2/100 of such variants might be associated with a high risk of cancer.

The posterior probability was calculated using Bayes factor analysis to combine the prior probability of pathogenicity and the combined odds of causality (Goldgar et al., 2008). Variants with combined odds for causality of ≤0.5 or ≥2.0 (Vallee et al., 2016) were considered to contain sufficient information to add to the prior probability of pathogenicity, and were classified based on the posterior probability following the IARC quantitative criteria as previously defined (Plon et al., 2008).

References

Atlas, E., Stramwasser, M., Whiskin, K., & Mueller, C. R. (2000). GA-binding protein alpha/beta is a critical regulator of the BRCA1 promoter. *Oncogene, 19*(15), 1933-1940. doi:10.1038/sj.onc.1203516

Auton, A., Brooks, L. D., Durbin, R. M., Garrison, E. P., Kang, H. M., Korbel, J. O., . . . Abecasis, G. R. (2015). A global reference for human genetic variation. *Nature, 526*(7571), 68-74. doi:10.1038/nature15393

Caminsky, N. G., Mucaki, E. J., Perri, A. M., Lu, R., Knoll, J. H., & Rogan, P. K. (2016). Prioritizing Variants in Complete Hereditary Breast and Ovarian Cancer Genes in Patients Lacking Known BRCA Mutations. *Hum Mutat, 37*(7), 640-652. doi:10.1002/humu.22972

Chang, T. H., Huang, H. Y., Hsu, J. B., Weng, S. L., Horng, J. T., & Huang, H. D. (2013). An enhanced computational platform for investigating the roles of regulatory RNA and for identifying functional RNA motifs. *BMC Bioinformatics, 14 Suppl 2*, S4. doi:10.1186/1471-2105-14-S2-S4

Davis, P. L., Miron, A., Andersen, L. M., Iglehart, J. D., & Marks, J. R. (1999). Isolation and initial characterization of the BRCA2 promoter. *Oncogene, 18*(44), 6000-6012. doi:10.1038/sj.onc.1202990

Dunham, I., Kundaje, A., Aldred, S. F., & Consortium, E. P. (2012). An integrated encyclopedia of DNA elements in the human genome. *Nature, 489*(7414), 57-74. doi:10.1038/nature11247

Easton, D. F., Deffenbaugh, A. M., Pruss, D., Frye, C., Wenstrup, R. J., Allen-Brady, K., . . . Goldgar, D. E. (2007). A systematic genetic assessment of 1,433 sequence variants of unknown clinical significance in the BRCA1 and BRCA2 breast cancer-predisposition genes. *Am J Hum Genet, 81*(5), 873-883. doi:10.1086/521032

Ernst, J., & Kellis, M. (2010). Discovery and characterization of chromatin states for systematic annotation of the human genome. *Nat Biotechnol, 28*(8), 817-825. doi:10.1038/nbt.1662

Fetzer, S., Tworek, H. A., Piver, M. S., & DiCioccio, R. A. (1999). Classification of IVS1-10T-->C as a polymorphism of BRCA1. *Cancer Genet Cytogenet, 113*(1), 58-64.

Goldgar, D. E., Easton, D. F., Byrnes, G. B., Spurdle, A. B., Iversen, E. S., Greenblatt, M. S., & Group, I. U. G. V. W. (2008). Genetic evidence and integration of various data sources for classifying uncertain variants into a single model. *Hum Mutat, 29*(11), 1265-1272. doi:10.1002/humu.20897

Kent, W. J., Sugnet, C. W., Furey, T. S., Roskin, K. M., Pringle, T. H., Zahler, A. M., & Haussler, D. (2002). The human genome browser at UCSC. *Genome Res, 12*(6), 996-1006. doi:10.1101/gr.229102

Lee, D., Gorkin, D. U., Baker, M., Strober, B. J., Asoni, A. L., McCallion, A. S., & Beer, M. A. (2015). A method to predict the impact of regulatory variants from DNA sequence. *Nat Genet, 47*(8), 955-961. doi:10.1038/ng.3331

Lek, M., Karczewski, K. J., Minikel, E. V., Samocha, K. E., Banks, E., Fennell, T., . . . Exome Aggregation, C. (2016). Analysis of protein-coding genetic variation in 60,706 humans. *Nature, 536*(7616), 285-291. doi:10.1038/nature19057

Lu, R., Mucaki, E. J., & Rogan, P. K. (2017). Discovery and validation of information theory-based transcription factor and cofactor binding site motifs. *Nucleic Acids Res, 45*(5), e27. doi:10.1093/nar/gkw1036

Mucaki, E. J., Caminsky, N. G., Perri, A. M., Lu, R., Laederach, A., Halvorsen, M., . . . Rogan, P. K. (2016). A unified analytic framework for prioritization of non-coding variants of uncertain significance in heritable breast and ovarian cancer. *BMC Med Genomics, 9*, 19. doi:10.1186/s12920-016-0178-5

Plon, S. E., Eccles, D. M., Easton, D., Foulkes, W. D., Genuardi, M., Greenblatt, M. S., . . . Group, I. U. G. V. W. (2008). Sequence variant classification and reporting: recommendations for improving the interpretation of cancer susceptibility genetic test results. *Hum Mutat, 29*(11), 1282-1291. doi:10.1002/humu.20880

Spurdle, A. B., Couch, F. J., Parsons, M. T., McGuffog, L., Barrowdale, D., Bolla, M. K., . . . kConFab, I. (2014). Refined histopathological predictors of BRCA1 and BRCA2 mutation status: a large-scale analysis of breast cancer characteristics from the BCAC, CIMBA, and ENIGMA consortia. *Breast Cancer Res, 16*(6), 3419. doi:10.1186/s13058-014-0474-y

Taberlay, P. C., Statham, A. L., Kelly, T. K., Clark, S. J., & Jones, P. A. (2014). Reconfiguration of nucleosome-depleted regions at distal regulatory elements accompanies DNA methylation of enhancers and insulators in cancer. *Genome Res, 24*(9), 1421-1432. doi:10.1101/gr.163485.113

Tavtigian, S. V., Byrnes, G. B., Goldgar, D. E., & Thomas, A. (2008). Classification of rare missense substitutions, using risk surfaces, with genetic- and molecular-epidemiology applications. *Hum Mutat, 29*(11), 1342-1354. doi:10.1002/humu.20896

Thompson, D., Easton, D. F., & Goldgar, D. E. (2003). A full-likelihood method for the evaluation of causality of sequence variants from family data. *Am J Hum Genet, 73*(3), 652-655. doi:10.1086/378100

Vallee, M. P., Di Sera, T. L., Nix, D. A., Paquette, A. M., Parsons, M. T., Bell, R., . . . Tavtigian, S. V. (2016). Adding In Silico Assessment of Potential Splice Aberration to the Integrated Evaluation of BRCA Gene Unclassified Variants. *Hum Mutat, 37*(7), 627-639. doi:10.1002/humu.22973
